# Investigating sand dunes flora conservation based on remote sensing and predictive modeling in the Mediterranean coastal region, Egypt

**DOI:** 10.1038/s41598-025-29710-y

**Published:** 2025-12-10

**Authors:** Mohamed M. El-Khalafy, Wael Mostafa, Yassin M. Al-Sodany, Kamal H. Shaltout, Soliman M. Toto

**Affiliations:** 1 Botany and Microbiology Department, Faculty of Science, Kafrelsheikh University, Kafrelsheikh, Egypt; 2 Geography Department, Faculty of Arts, Kafrelsheikh University, Kafrelsheikh, Egypt; 3 Botany and Microbiology Department, Faculty of Science, Tanta University, Tanta, Egypt; 4 Botany and Microbiology Department, Faculty of Science, Alexandria University, Alexandria, Egypt

**Keywords:** Remote sensing, SDM, Prediction, Sand dunes, Vegetation, Mediterranean, Conservation, Climate sciences, Ecology, Ecology, Environmental sciences

## Abstract

Climate change and anthropogenic activities present significant long-term threats to global biodiversity. To effectively reduce species loss, conservation strategies must rely on detailed insights into suitable habitats for different species and the factors influencing their distribution. This research aimed to provide a checklist of the plant species on the Mediterranean coastal sand dunes in Egypt and investigated the potential impact of anthropogenic and climate change on its distribution in Egypt over the next several decades using remote sensing and species distribution modeling (SDM). The current study revealed that Egypt’s Mediterranean coastal sand dunes host 236 plant taxa, comprising 212 species, 5 varieties, and 19 subspecies, distributed across 75 genera and 39 families. An ensemble modeling approach was employed, incorporating five key. Among these, Random Forest (RF), Boosted Regression Trees (BRT) demonstrated the highest performance. For remote sensing, Landsat imagery (including Thematic Mapper (TM), and Operational Land Imager (OLI) sensors) was utilized. Support Vector Machine (SVM) classification, implemented via Google Earth Engine (GEE), was employed to analyze land use/cover changes. Additionally, future land use projections for 2080 were generated using the CA-Markov simulation model. The study detected major changes in land use along the Mediterranean coast from 1988 to 2024, with projections extending to 2050 and 2070. Based on the ensemble model, the primary environmental drivers of sand dune vegetation were distance from the shoreline, soil organic carbon, precipitation patterns (both total in the wettest month and seasonality), and sand content. The landscape was significantly transformed by the expansion of urban areas, agriculture, and aquaculture. Vegetation diversity is abundant in the Eastern and Western Mediterranean dunes, but scarce in the Deltaic Mediterranean dunes. Future projections using the IPSL-CM6A-LR GCM model for 2050 and 2070, under two climate scenarios (sustainable and low-emissions future: SSP126 and very high greenhouse gas emission: SSP585), suggested a decline in suitable habitat compared to current conditions. These findings emphasize the pressing need for conservation actions, including species reintroduction and strategic planning for both in situ and ex situ conservation in viable habitats.

**Supplementary Information:**

The online version contains supplementary material available at 10.1038/s41598-025-29710-y.

## Introduction

Coastal Mediterranean sand dunes are ecologically vital transition zones between land and sea that host unique biodiversity and provide essential ecosystem services, yet they are among the most threatened habitats globally due to intense human pressure^1–3^. These ecosystems support a highly specialized flora and fauna adapted to the harsh sea-inland environmental gradient, with vegetation playing a critical bio-geomorphological role by trapping sand to build and maintain the dune structure itself^4^. In the Mediterranean Basin, the coastal sand dunes play a crucial role in supporting biodiversity, fostering environmental diversity, and providing varied habitats. Furthermore, well-preserved dunes serve as the first natural barrier against coastal erosion, protecting inland areas from the destructive forces of wind and storm waves^5^. Beyond their intrinsic natural value, these dunes deliver indispensable benefits to society, including coastal protection from storms and erosion, groundwater storage, climate regulation through carbon sequestration, and opportunities for recreation and tourism^1^.

Globally, parabolic coastal sand dunes are rare and found only in specific regions, including Egypt, Brazil, New Zealand, Saudi Arabia, Australia, South Africa, and the United States^6^. In contrast, along the Mediterranean coast from Egypt westwards to Morocco, sand dunes run parallel to the shoreline, spanning widths from a few meters to 10 km and dating back to the Pleistocene Era^7^. The western Mediterranean coast, extending from Alexandria to Matruh, features distinctive oolitic sand dunes that form parallel to the sea^8^. These dunes, located just south of the shore, can reach heights of up to 10 m and stretch 0.5–1.5 km inland. Covering nearly 900 km, much of this coastline is lined with diverse sand dune formations. These dunes create a unique landscape with specialized flora and serve as a critical stopover for migratory birds, such as quails migrating from Europe.

Egypt’s Mediterranean coastal dunes have experienced a dramatic loss of 75% of their area in recent decades due to human-driven environmental degradation^9^, resulting in severe ecological consequences such as habitat destruction, loss of economically important species, and reduced genetic biodiversity. The unique sand-adapted plant communities are under threat from overuse, pollution, rising sea levels, overgrazing, and urban expansion. These plants cannot survive further south due to climatic constraints^10^, specific soil requirements, and increased competition from generalist species in more arid environments^7,11^. The coastal dunes’ unique white calcareous sand provides essential conditions for these plants^12–14^, which are further endangered by urbanization and climate change, leading to a decline in plant diversity and richness. The coastal dune flora includes many endemic and highly specialized species that thrive in low-competition environments. Moving south, they would encounter greater competition from generalist desert shrubs and grasses that are better adapted to those biotic and abiotic conditions, ultimately outcompeting the dune specialists^15^. Recently, urbanization and climate change have further endangered these habitats, leading to a decline in plant diversity and richness^16^.

Over the past two decades, Egypt’s socio-economic changes and open-door economic policies have spurred extensive development along the coastal dune belt west of Alexandria. Large stretches of these dunes have been destroyed to make way for summer resorts, built within 100 m of the shore and extending 400–600 m inland, with widths of 400 m or more along the coastline. Much of this area has already been replaced by buildings, gardens, and infrastructure^12^. According to^17] and [18^, ecosystem degradation has been driven by overgrazing, excessive plant collection, deforestation, agricultural expansion, resort construction, industrial growth, vehicle disturbances, and quarrying. There is an urgent need for management strategies to mitigate coastal degradation through combined local and global efforts^19^. A major factor in the ecosystem decline is the destruction of plant cover, which helps control runoff and wind erosion^20^. Ahmed et al.^21^ identified 66 plant species at risk due specifically to resort construction and urged an immediate halt to dune destruction for resorts and other land uses along the Mediterranean coast. Moreover, the growth of urban areas has caused major shifts in land use and land cover, significantly altering temperature patterns^22,23^. This process, referred to as the urban heat island effect, results in substantially higher temperatures in urban zones compared to surrounding rural areas. Consequently, analyzing the connection between human activities, urban expansion, and rising temperatures is essential for predictive modeling and solution development in coastal dune ecosystems^24^.

Climate change has triggered rising temperatures and more frequent drought events across global regions^25^. In the Mediterranean basin and its sub-regions, this trend manifests through steadily increasing air and sea surface temperatures, along with anticipated shifts in precipitation patterns^26^. When these climatic alterations interact with anthropogenic pressures (land use changes, pollution, and resource overexploitation), they risk exceeding ecosystem resilience thresholds^25^, ultimately transforming ecological structures and functions^27^. Such environmental modifications may significantly influence plant species distributions by altering thermal and hydrological conditions^28^. Climate change-induced extreme weather events, particularly droughts and heat waves, often significantly affect plant communities and ecosystems^29^. These ecological impacts primarily stem from differences in species’ adaptive capacities and functional traits, which determine their varying responses to environmental stressors^29^. Several Mediterranean coastal dune species and habitats are highly vulnerable due to their specialized ecological roles and acute sensitivity to human pressure. Across Italy, the conservation status of the shifting dune habitat formed by marram grass (*Ammophila arenaria*) is poor and classified as critically endangered. As the primary engineer of the foredune, this plant community is severely threatened by pressures from erosion, urbanization, and trampling^2^. Morever, overgrazing, over-cutting, and habitat fragmentation are diminishing populations of *Periploca angustifolia* on the stabilized back dunes of Egypt’s northwestern Mediterranean desert^14^. Furthermore, the expansion of tourism infrastructure and coastal development has led to the local extinction of several unique species, such as the emblematic *Anchusa* (Boraginaceae) in the Corso-Sardinian region and the highly threatened *Stachys maritima* (Lamiaceae) in the western Mediterranean^30^.

Over the past few decades, the field of land change science has undergone significant development. A key aspect of this discipline involves observing, monitoring, and characterizing land transformations, where remote sensing has been a critical tool^31^. Land Change Science (LCS) is a critical trans-disciplinary field that aims to observe, monitor, and understand the dynamics of human-modified landscapes, making it essential for addressing global environmental challenges. Its importance lies in its integrated approach to quantifying the causes, consequences, and feedbacks of land-use and land-cover change (LULCC), which is a primary driver of biodiversity loss, climate change, and the alteration of ecosystem services. For instance, LCS research has been pivotal in documenting the global expansion of agriculture and urban areas as dominant processes transforming the Earth’s surface, leading to significant carbon emissions and habitat fragmentation. By leveraging remote sensing, geographic information systems (GIS), and socio-economic data, LCS provides the empirical evidence base necessary for informed land-use planning, natural resource management, and policy development, from local conservation efforts to international agreements like the UN Sustainable Development Goal^32,33^.

Researchers have employed satellite remote sensing and GIS technologies to assess and forecast land use and land cover changes^34–36^. Machine learning techniques like Random Forest, Support Vector Machine (SVM), and Artificial Neural Networks (ANN) are gaining prominence in land cover classification due to their ability to recognize and adapt to intricate land cover patterns, enhancing classification accuracy. Nevertheless, conventional approaches such as maximum likelihood classification remain popular for their simplicity and ease of implementation^37,38^.

The application of SDMs to forecast the impacts of specific pressures, such as urban expansion and rising temperatures, is essential for formulating effective strategies to mitigate the effects of climate change^39^. These models are particularly valuable for predicting alterations in species’ geographical ranges across varying climatic conditions. By incorporating multiple environmental parameters, including bioclimatic, topographic, edaphic, and habitat variables, SDMs can reliably characterize species’ ecological niches and forecast potential distribution changes under different climate projections^40^.

Research strongly advocates for ensemble modeling approaches over single-model methods when assessing climate-driven species range shifts^41,42^. These techniques, which integrate outputs from multiple models, offer superior performance in several key aspects: they enhance result reliability and accuracy while preventing model overfitting^41^. Particularly valuable for rare species distribution modeling, ensemble approaches significantly reduce prediction errors and generalization limitations. When projecting future climate impacts on biodiversity at scale, ensemble modeling proves particularly advantageous, making it the preferred methodology for studies involving numerous species^43^. Moreover, Ensemble modeling is widely recognized for reducing predictive uncertainty and improving model robustness, while effectively preventing overfitting^44–46^.

Given the threats of pollution, overgrazing by camels and sheep, and rapid urbanization along Egypt’s Mediterranean coast, assessing the current state of these ecosystems is essential. The research will evaluate the distribution of the vegetation in the coastal Mediterranean sand dunes in Egypt to understand how environmental changes influence this habitat. The present study establishes its priority by comprehensively investigating the vegetation of sand dune habitats across the entire Mediterranean coastal strip of Egypt. Its primary novelty lies in the application of Species Distribution Models (SDMs) integrated with remote sensing data, combining spectral indices with traditional environmental variables to build superior predictive models for key native species. This integrated approach not only assesses the current status of the coastal dune vegetation but also projects its expected future distribution under forthcoming environmental conditions, providing a critical, forward-looking perspective on the dynamics of this vulnerable ecosystem. Previous investigations into Mediterranean coastal dunes have largely been confined to the western sector, leaving a gap in comprehensive data for other areas. Species distribution modeling and remote sensing, along with available environmental predictors (bioclimatic and soil parameters), will be utilized to: (1) provide comprehensive assessment of vegetation distribution in Egypt’s Mediterranean dunes under changing environmental conditions, (2) identify the key factors influencing the vegetation composition and distribution in the coastal Mediterranean sand dunes in Egypt, (3) map and anticipate its possible range under present and upcoming climate scenarios, and (4) evaluate the impact of climate fluctuations and anthropogenic activities on its prospective distribution utilizing the general circulation model IPSL-CM6ALR.

## Materials and methods

### Study area

North Egypt encompasses a highly diverse region, stretching along approximately 1000 km of Mediterranean coastline. The Mediterranean coastline in Egypt can be classified into three distinct sectors: the eastern (240 km from Rafah to Port-Said), middle (180 km from Port-Said to Alexandria), and western (550 km from Alexandria to Sallum), differentiated by their soil characteristics, rainfall patterns, and vegetation types^8^. The three sectors of Egypt’s Mediterranean coast are primarily distinguished by a strong west-to-east aridity gradient, which directly influences their ecological character. The western sector, from Alexandria to Sallum, receives the highest rainfall, fostering the most developed and stable sand dunes with the richest diversity of native flora. In contrast, the eastern sector, from Rafah to Port-Said, is the most arid, resulting in saline, sandy soils and vegetation limited to sparse, drought- and salt-tolerant species. The middle sector, situated between Port-Said and Alexandria, acts as a transitional area with moderate rainfall but has been the most extensively altered by human activities, with its natural vegetation largely supplanted by agriculture and urban expansion^8^.

**Fig. 1:**
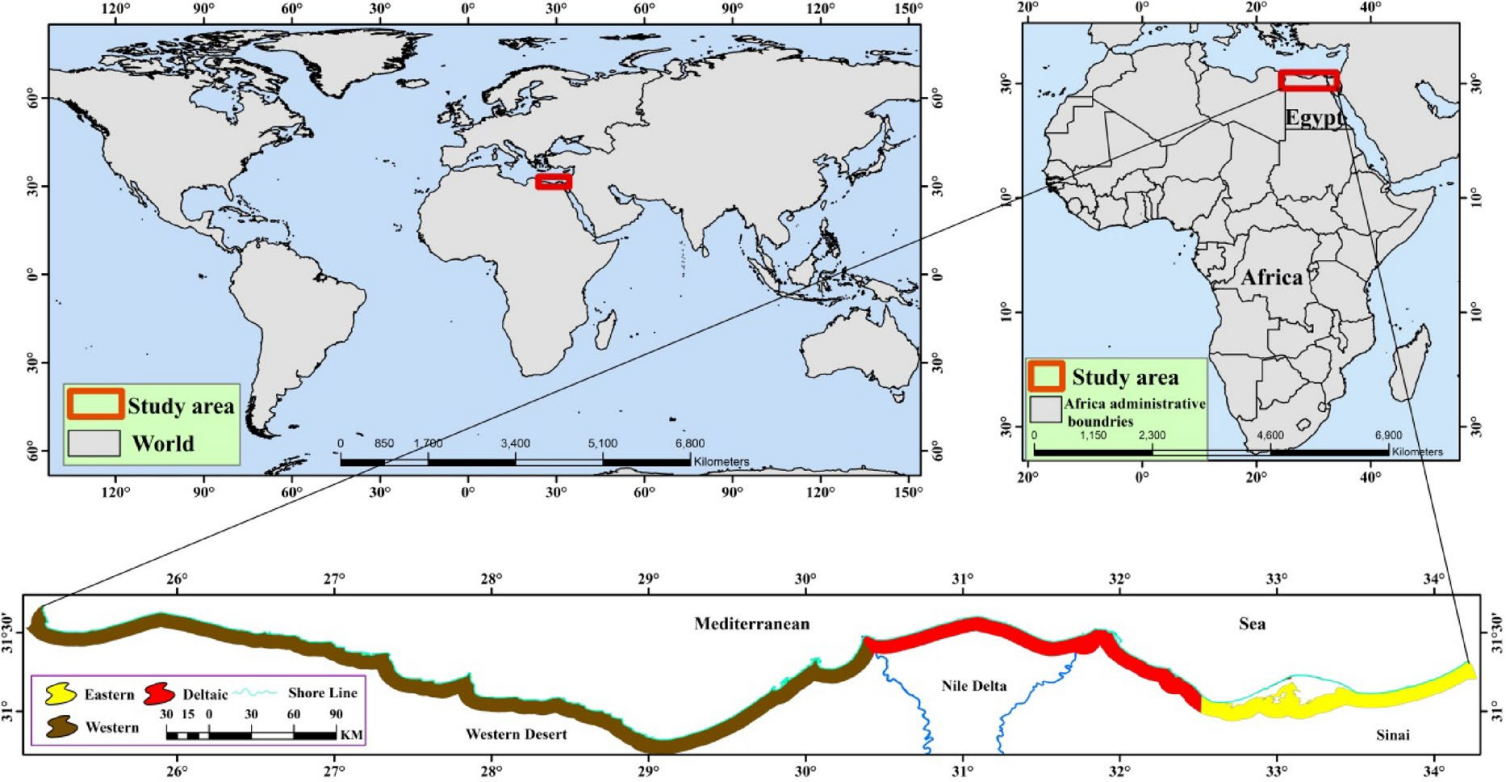
Map showing the Mediterranean coastal strip in Egypt.

Notably, the western coast exhibits the highest floral diversity with 968 vascular plant species^47,48^ and is predominantly bordered by diverse sand dune systems of varying formations and ecological characteristics^12,21^. The Mediterranean Coast is comparatively large, and includes sand dunes, lakes and lagoons, deltaic sediments, mud flats, plateaus and rocky beaches^49^ (Fig. 1).

### Checklist and floristic analysis

From spring 2020 to winter 2024, several field expeditions were conducted throughout Egypt to survey the vegetation in the coastal Mediterranean sand dunes in Egypt. During these surveys, plant specimens were collected from wherever possible, along with observations on reproductive forms, life forms, and flowering periods. The present study documented essential ecological data including habitat characteristics, GPS coordinates, human uses, and conservation threats for each taxon. The field studies on plants, including the collection of plant material, comply with relevant institutional, national, and international guidelines and legislation. Plant material identification was performed by Prof. Dr. Kamal H. Shaltout, Botany Department, College of Science, Tanta University. The gaps were compiled from six major herbarium collections: Tanta University Herbarium (TANE), Cairo University Herbarium (CAI), Herbarium of Agriculture Museum (CAIM), Desert Research Center Herbarium (CAIH), National Research Centre Herbarium (CAIRC) and Alexandria University Herbarium (ALEX), and through comprehensive review of published literature encompassing journal articles, reference books, graduate theses, and technical reports:^12,16,20,21,50–53^. The plant checklist was organized alphabetically following the Angiosperm Phylogeny Group (APG) IV classification system, with taxonomic verification using multiple authoritative sources (e.g., global databases, regional floras, specialist literature and herbarium records). Scientific names were cross-checked against Plants of the World Online database (POWO). The plant life forms in this study were categorized using Raunkiaer^54^ classification framework, following modifications proposed by Kershaw^55^. Goods of the natural flora in the ecosystem include species and their parts and products that grow in the wild and are used directly for human benefit^56^; while ecosystem services are those valuable, ongoing streams of benefits provided by thriving ecosystems^57^. Goods are classified into 6 major categories: grazing, medicinal, human food, timber, fuel, and other uses (e.g. making mats, baskets, chairs, ornamental uses, beach bed, soap manufacture, oil and dye extraction scientific studies and historical values).

We analyzed the phytogeographical distribution of the vegetation in the coastal Mediterranean sand dunes through field surveys and herbarium examinations, depending on Boulos^52^. The global distribution (chorotype) patterns of the documented plants were evaluated using Good floristic classification system^58^, which organizes the world’s vegetation into six major kingdoms, three subkingdoms, and thirty-nine distinct floristic regions. The IUNC categories were provided according to IUCN categories and criteria (https://www.iucnredlist.org/*).*

### Vegetation sampling

To define the current floristic composition and structure of Egypt’s Mediterranean coastal dunes, a comprehensive field survey was carried out between 2020 and 2024, coinciding with the peak flowering time to ensure maximum species detection (April), June and November in each year. A stratified random sampling design was used to capture the whole ecological variety across three major dune systems: foredunes, mobile dunes, and consolidated dunes along the western, deltaic, and eastern coastal sectors. This technique reduced spatial bias while capturing microhabitat and regional heterogeneity in vegetation assemblages.

A total of 346 quadrats (each 50 × 50 m) were systematically established across 140 representative sites (Sallum, Matrouh, Ras El-Hekma, El-Dabaa, Sidi Abdel Rahamn, Alamin, El-Hammam, Idku, Rashid, Baltim, Port Said, Rafah, Al-Arish), with proportional allocation among dune types based on their areal extent and vegetation complexity. The quadrates were designed to include the majority of plant species in the selected area. Within each quadrat, all vascular plant taxa were recorded, and their cover-abundance was visually estimated using the modified Braun-Blanquet scale^59^, providing a robust semi-quantitative measure of species dominance and frequency.

### Remote sensing

#### Land use/cover classification

Remote sensing is fundamentally transformative for studying Mediterranean coastal dune vegetation, as it enables the efficient, large-scale, and multi-temporal analysis of these dynamic and often inaccessible ecosystems^60^. This research analyzed land cover projections and historical transformations in northern Egypt using multitemporal satellite data from 1988 to 2080. The study utilized eight Landsat-5 Thematic Mapper (TM) images from June 1988 and Landsat-8 Operational Land Imager (OLI) data from 2024, both sourced from the USGS and resampled to 30-meter resolution (Table 1)^61^. Landsat-9 OLI data provides calibrated, medium-resolution imagery that is fundamental for long-term Earth observation, including applications like monitoring agricultural health, mapping deforestation, managing water resources, and, crucially for our research, tracking changes in coastal ecosystems like Mediterranean sand dunes^61^. All images were processed with radiometric and atmospheric corrections. The radiometric and atmospheric corrections applied to Landsat imagery are essential processing steps that convert raw digital numbers into accurate surface reflectance values by removing distortions from sensor sensitivity and atmospheric interference. Radiometric correction addresses sensor-specific variations by converting raw digital numbers to top-of-atmosphere (TOA) reflectance. This process involves using calibration coefficients to account for sensor degradation and solar illumination angles. Atmospheric correction then removes the scattering and absorption effects caused by gases and aerosols to derive surface reflectance^62^. Land cover classification was conducted using Support Vector Machine (SVM) algorithms within Google Earth Engine (GEE), leveraging its capacity for handling large-scale geospatial datasets. The SVM-GEE integration is powerful as it is able to identify vegetation and urbanization trends while enabling long-term projections up to 2080 under regional climatic and environmental conditions^61^.

**Table 1:** The two Landsats utilized for obtaining remote sensing images from 1988–2024 (https://earthexplorer.usgs.gov/).

Satellite	Sensor	Band name	Band width (μm)	Resolution (m)	Path row	Acquisition date
Landsat 5	Thematic mapper (TM)	Band 1 Blue	0.45–0.52	30	174/39 175/39 176/39 177/39 178/39 179/39 180/39 181/39	June 1988
		Band 2 Green	0.52–0.60	30		
		Band 3 Red	0.63–0.69	30		
		Band 4 NIR	0.77–0.90	30		
		Band 5 SWIR 1	1.55–1.75	30		
		Band 6- Thermal Infrared (TIRS) 1	10.40–12.50	120		
		Band 7- SWIR 2	2.08–2.35	30		
Landsat 8	Operational land imager (OLI)	Band 1 Coastal	0.43–0.45	30	174/39 175/39 176/39 177/39 178/39 179/39 180/39 181/39	June 2024
		Band 2 Blue	0.45–0.51	30		
		Band 3 Green	0.53–0.59	30		
		Band 4 Red	0.64–0.67	30		
		Band 5 NIR	0.85–0.88	30		
		Band 6 SWIR 1	1.57–1.65	30		
		Band 7 SWIR 2	2.11–2.29	30		
		Band 8 Pan	0.50–0.68	15		
		Band 9 Cirrus	1.36–1.38	30		
		Band 10 TIRS 1	10.6–11.9	100		
		Band 11 TIRS 2	11.5-12.51	100		

#### Cellular automata (CA) Markov model

The Cellular Automata (CA) Markov Model is a hybrid simulation tool that projects future land-use changes by combining the quantitative predictive power of Markov chains, which determine the amount of change between categories, with the spatial allocation rules of Cellular Automata, which define where changes are likely to occur based on neighborhood relationships. This integration generates realistic, spatially explicit projections of future land-use patterns. This study utilized IDRISI Selva 17 software to forecast land cover changes for 2041–2060 and 2061–2080 through the CA-Markov model, following Anderson’s standardized classification framework for remote sensing-based land use/land cover analysis^63^. The two techniques used to calculate the transition probability matrix between land cover categories from 1988 to 2024 based on high-resolution SVM classification maps, and a CA-Markov model, which integrates these Markov probabilities with Cellular Automata spatial simulation to realistically simulate the future spatial distribution of changes. Using this calibrated model, predictive maps were generated to illustrate the projected distribution of land cover in the study area for the years 2050 and 2070. After that the predictive raster was incorpoted into R Software with other climate parameters to obtain the predivtive future maps. We customized this established methodology to align with regional characteristics and study requirements^64^, incorporating multiple land cover categories specific to the area. Our customization of the CA-Markov model specifically addressed the unique Mediterranean coastal dune environment of Egypt by incorporating regionally-specific land cover categories such as “bare sand,” “pioneer dune vegetation”, and “stable dune shrubland”.We integrated key environmental drivers including the west-to-east aridity gradient, soil organic carbon content, distance from shoreline, and anthropogenic pressure from urban areas and roads into the Cellular Automata transition rules. This approach enabled our model to accurately simulate ecologically relevant processes like dune succession and coastal squeeze rather than just generic land use changes. The CA-Markov model is a hybrid land-change simulation model that integrates a Markov chain for predicting the quantitative amount of land-use change and CA for allocating the spatial location of these changes^65^. The software proved highly effective in managing extensive datasets and producing precise projections critical to the investigation. Beyond the core CA-Markov analysis, the prediction reliability was enhanced through complementary approaches: acquisition of up-to-date satellite imagery and implementation of refined image processing algorithms^66^.

### Species distribution modeling (SDM)

#### Species records collection

**Fig. 2:**
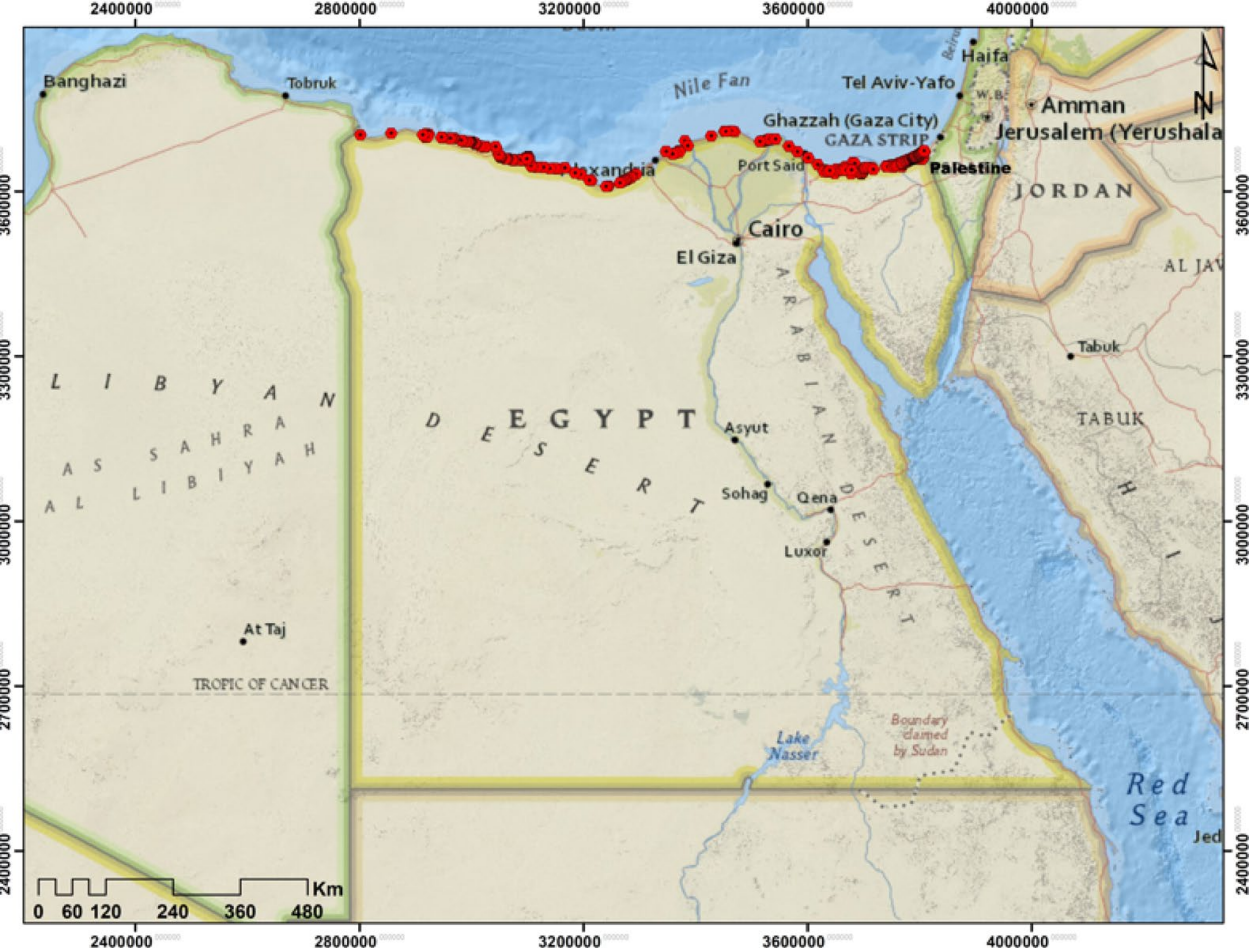
Location map of the study area with current occurrence points (red circles) of the vegetation in the Mediterranean coastal sand dunes in Egypt.

We compiled a database of recent georeferenced occurrence records for the vegetation in the Mediterranean coastal sand dunes in Egypt, gathering a total of 572 records from field trips (439 points), published sources (62 points) and online platforms such as GBIF (http://www.gbif.org/*)* (71 points) (Fig. 2).

To reduce the impact of sampling bias^67^, we applied a spatial filtering approach, retaining only one randomly selected record per 2.5 × 2.5 arcmin grid cell (equivalent to 4.6 × 4.6 km at the equator), matching the resolution of our environmental predictors. Records with evident geocoding inaccuracies were excluded, and duplicates were manually removed. The final dataset consisted of 420 spatially unique occurrence records (346 from filed trips, 34 from published sources and 40 from online platforms), distributed across the Mediterranean coastal sand dunes in Egypt, meeting the minimum sample size recommended for reliable model calibration^68^. While we recognize that the limited sample size may influence the robustness of the species distribution model (SDM), prior research suggests that SDMs based on small datasets can still yield meaningful predictions^69,70^. A small sample size can significantly compromise the accuracy of Species Distribution Models (SDMs) by reducing statistical power, increasing model variance, and heightening the risk of overfitting, where models perform well on training data but fail to generalize to new areas, ultimately leading to unreliable habitat suitability maps and inflated performance metrics^71,72^. Furthermore, we used algorithms like MaxEnt known for their relative robustness with small sample sizes and employed leave-one-out cross-validation to obtain more realistic performance estimates than standard validation methods would allow with limited data^73^.

#### Environmental predictors

The climatic variables analyzed included total annual precipitation, maximum, minimum, and average temperatures, wind speed, solar radiation, water vapor pressure, and 19 bioclimatic variables. These were obtained from the WorldClim Database^74^; WorldClim v2.1), representing averages for 1970–2000 (Appendix 1). Topographic features were represented by elevation data, along with derived slope and aspect measurements. All variables had a spatial resolution of 30 arc-seconds. Elevation data were sourced from the U.S. Geological Survey (https://www.usgs.gov), while slope and aspect were computed using ArcGIS v10.8. Edaphic factors were computed from SoilGrids^75^; https://soilgrids.org). The human footprint map, the most widely used indicator of human influence, is calculated by combining several key factors: population density, built-up infrastructure (like roads and factories), ease of access to an area, and electrical energy use (measured by nighttime lights). The map was sourced from hub ArcGIS (https://hub.arcgis.com). The datasets were clipped to the study area’s boundaries using the same software.

To minimize redundancy, a correlation analysis based on variance inflation factors (VIFs) was conducted to assess multicollinearity among explanatory variables. Highly correlated variables (VIF ≥ 5) were excluded due to their negligible contribution^76^. The VIF analysis was performed in R 4.2.2 using the *usdm* package^77^, specifically with the vifcor and vifstep functions^78^.

For future projections under climate change scenarios, we utilized data from one General Circulation Model (GCM) (IPSL-CM6A-LR) (spatial resolution of 30 arc-seconds) obtained from WorldClim 2.1 (www.worldclim.com*).* CMIP6 (Coupled Model Intercomparison Project Phase 6) models are the latest generation of global climate models developed by an international scientific collaboration to understand past, present, and future climate changes, forming the scientific basis for reports like the IPCC’s Sixth Assessment Report. These models simulate the physics, chemistry, and biology of the Earth’s system but at a native resolution that is often too coarse for regional or local impact studies^79^. These downscaled CMIP6 models were selected for their accurate representation of current climate conditions and their reliable simulation of global and regional temperature and precipitation patterns. They are particularly effective in projecting global warming trends and long-term variations in climatic variables, predicting a more pronounced temperature increase compared to other models^80,81^. The IPCC’s Sixth Assessment Report (AR6) processed temperature and precipitation projections from 23 GCMs across four Shared Socioeconomic Pathways (SSPs: 1–2.6, 2–4.5, 3–7.0, and 5–8.5), representing varying emission scenarios. In this study, future species distribution modeling was conducted for IPSL-CM6A-LR over three timeframes: the 2050s (2041–2060 average), and the 2070s (2061–2080 average), under two SSPs (SSP1-2.6 and SSP5-8.5).

#### Modeling procedure

An ensemble of five modeling algorithms was used to construct a robust species distribution model (SDM). The selected methods included: Generalized Linear Model (GLM:^82^ as parametric approach, Boosted Regression Trees (BRT:^83,84^, Random Forest (RF:^85,86^ as non-parametric machine learning techniques, and Support Vector Machines (SVM:^87–89^) as machine learning method effective for binary classification in ecological modeling and Maxent (Maximum Entropy). These models were chosen for their stability, transferability^90–92^, and strong performance in cross-validation and external validation^43^. Models were built by averaging the results of the five algorithms (ensemble models). It was employed to enhance predictive accuracy by mitigating the limitations of individual models^84^. The primary reason for using this particular combination is that it leverages the strengths of each algorithm type while mitigating their individual weaknesses. Parametric models like GLM offer high interpretability and a check against over-complexity, while the machine learning methods (BRT, RF, SVM) excel at capturing the complex, non-linear relationships between species and their environment that are common in nature. MaxEnt is included as it is arguably the most widely used and optimized algorithm for presence-only data, a common data type in ecology. By combining their predictions into an ensemble, the model avoids over-reliance on any single method, resulting in a more accurate, stable, and generalizable projection of species distribution that is less sensitive to the specific biases of any one algorithm^76,93,94^.

Modeling was performed using the sdm package (v1.1-8) in R 4.2.3^77^. Species distribution under current climatic conditions was predicted using 70% of the data for training and 30% for testing and evaluation^91^. The ensemble model (EM) was weighted based on the True Skill Statistic (TSS) and the Maximum Training Sensitivity plus Specificity (MTSS) threshold, which minimizes both over- and under-prediction errors^76,95^. TSS is a threshold-dependent performance metric for species distribution models that balances Sensitivity (the ability to correctly predict presences) and Specificity (the ability to correctly predict absences), calculated as (Sensitivity + Specificity − 1) and ranging from − 1 (worse than random) to + 1 (perfect agreement), with values above 0.7 generally indicating good to excellent model performance. MTSS is not a metric but a specific threshold value; it is the optimal cut-off point on the model’s probability output (e.g., habitat suitability from 0 to 1) that, when used to convert probabilities into a binary presence-absence map, maximizes the TSS value, thereby providing an objective and balanced method for this critical conversion in model application^96,97^.

The final ensemble output was a logistic suitability map, with values ranging from 0 (unsuitable) to 1 (highly suitable). For further analysis, habitat suitability was classified into three categories (low, medium, high) in ArcGIS 10.8.2^98–100^. Changes in the predicted ecological extent of the studied species between the current and future climatic scenarios in correspondence with classes were computed as follows: ensemble model of current and future habitat suitability projections were converted into binary maps (presence/absence) based on the MTSS threshold. Afterward, we applied the equation (Future prediction*2) - (current prediction) using raster calculator option in spatial analyst tool in Arc Toolbox, to estimate the changes. The resulting output was then visualized as loss, stable, and gain areas in ArcGIS 10.8.2 as detailed in Dakhil^101^.

#### Model evaluation

Our study provides a comprehensive assessment of anthropogenic and ecological impacts on the vegetation colonization and distribution patterns across the Mediterranean coastal sand dunes in Egypt. Utilizing advanced species distribution modeling (SDM) techniques, we predicted potential range shifts under changing climatic conditions. The ensemble modeling approach demonstrated exceptional predictive performance, as confirmed by multiple robust evaluation metrics. The carefully selected environmental predictors generated a highly reliable model, evidenced by consistently high AUC and True Skill Statistic (TSS) scores, along with other performance indicators. This strong predictive capacity effectively captures the relationship between the environmental drivers and the Mediterranean coastal sand dunes vegetation together with species distribution in Egypt, as validated against independent field data.

To assess model performance, we employed multiple accuracy metrics, including overall accuracy, sensitivity, specificity, and the True Skill Statistic (TSS). TSS values range from − 1 to + 1, with + 1 indicating perfect predictive ability and values ≤ 0 suggesting performance no better than random. Additionally, we evaluated model accuracy using the Area Under the Curve (AUC) of the Receiver Operating Characteristic (ROC). The AUC is a widely used metric for assessing species distribution models (SDMs) due to its independence from threshold selection^102–104^. An AUC value closer to 1 indicates superior model performance^94,105^. The ROC curve was generated by plotting the true positive rate (sensitivity) against the false positive rate (1 − specificity)^106^.

## Results

### Checklist of mediterranean coastal sand dunes in Egypt

**Fig. 3:**
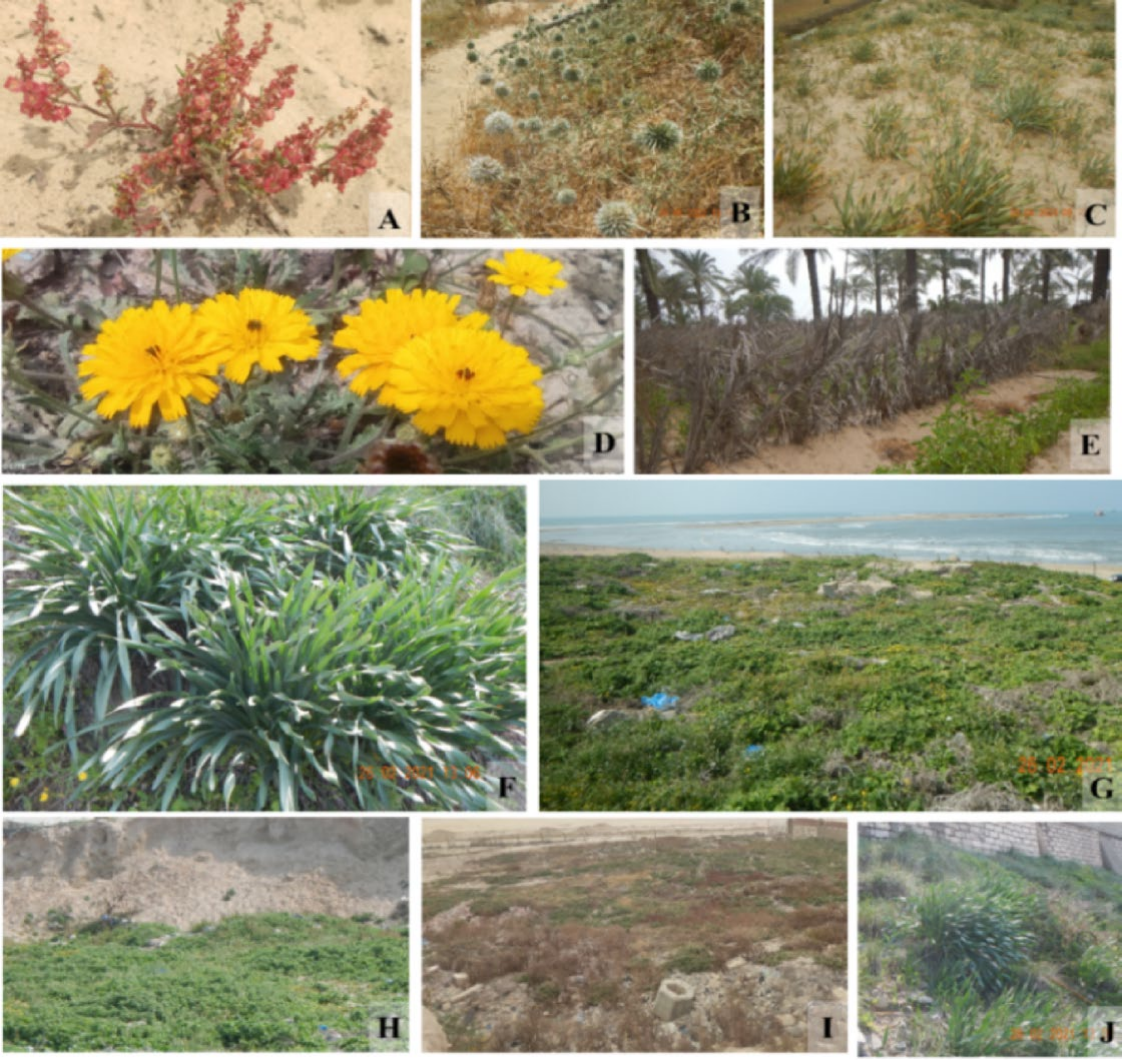
Examples of important plants and threats along the Mediterranean coastal strip in Egypt. (**A**) *Rumex pictus* Forssk., (**B**) *Echinops spinosissimus* Turra, (**C**) *Pancratium maritimum* L., (**D**) *Picris asplenioides* L., (**E**) Agricultural practice in coastal sandy dunes, (**F**) *Pancratium maritimum* L., (**G**) *Mesembryanthemum crystallinum* L., (**H**) Habitat loss due to sand transfer, (**I**) Solid waste and demolition, (**J**) Urbanization. From (**A**) to (**E**), the Edku site, from (**F**) to (**J**) Abi Qir site (taken by Soliman M. Toto, Mohamed M. El-Khalafy and Yassin M. Al-Sodany).

**Fig. 4:**
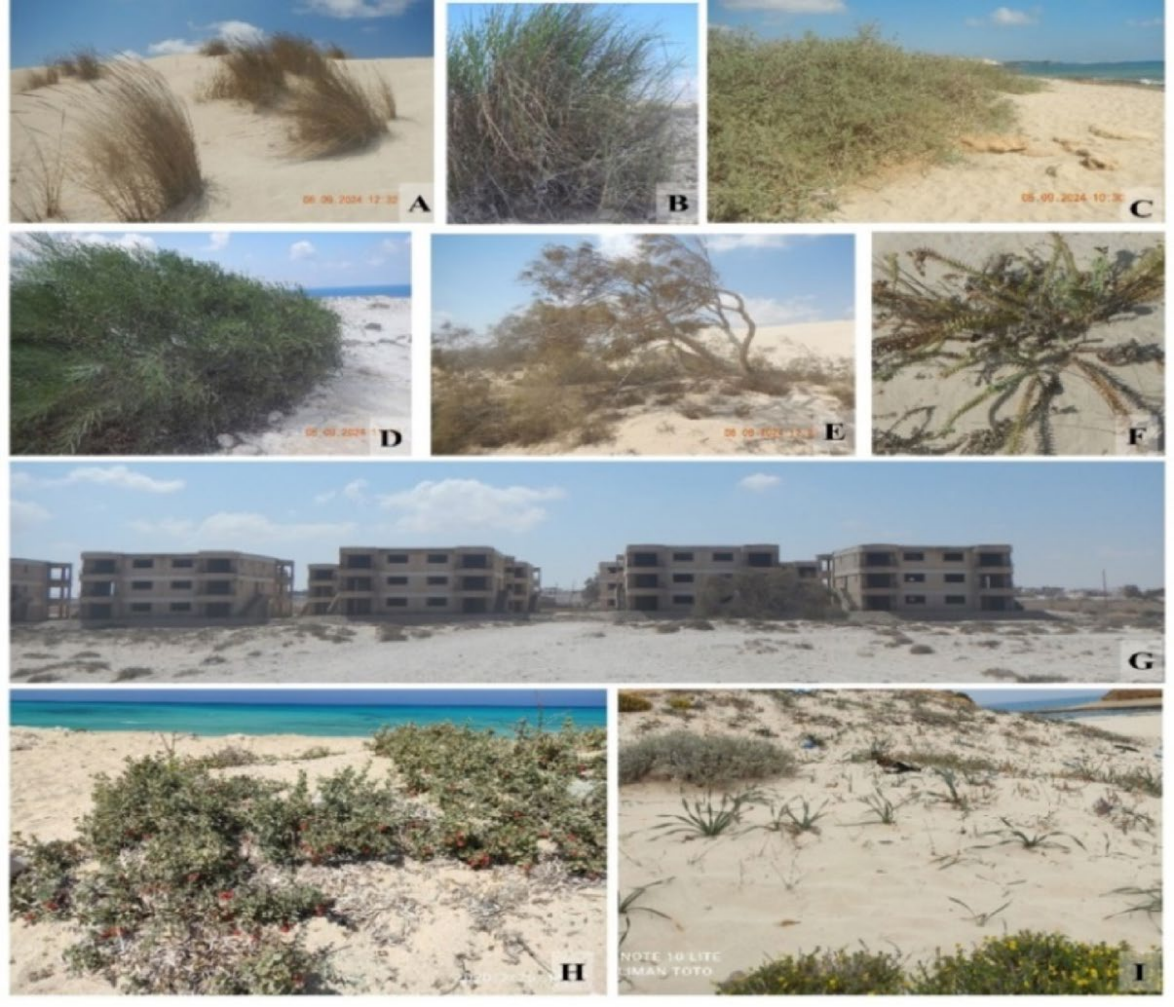
Examples of important plants and threats along the Mediterranean coastal strip in Egypt. (**A**) *Calamagrostis arenaria* (L.) Roth, (**B**) *Saccharum spontaneum* L., (**C**) *Nitraria retusa* (Forssk.) Asch., (**D**) *Acacia saligna* (Labill.) H.L.Wendl., (**E**) *Tamarix aphylla* (L.) H.Karst., (**F**) *Euphorbia paralias* L., (**G**) Urbanization and tourist resort building, (**H**) *Nitraria retusa* (Forssk.) Asch., (**I**) *Ononis vaginalis* Vahl, *Pancratium maritimum* L. From A to G Ras El Hekma. H El-Alamein site. I Marsa Matruh site (taken by Soliman M. Toto, Mohamed M. El-Khalafy and Yassin M. Al-Sodany).

The present study indicated that the Mediterranean coastal sand dunes in Egypt includes 236 taxa (212 species, 5 varieties and 19 subspecies) belonging to 75 genera and 39 families (Appendix 2 and Figs. 3 and 4). Among them, 34 taxa are restricted to sand dunes habitat. *Zygophyllum* is the most represented genus (6 taxa), then *Lotus* and *Plantago* (each of 5 taxa), followed by *Centaurea*, *Launea* and *Allium* (each of 4 taxa). The most represented families are Asteraceae (51 taxa), followed by Poaceae (27 taxa), Fabaceae (25 taxa), and Amaranthaceae (18 taxa), while eleven families are represented by one taxon only (Appendix 2). Regarding the national phytogeographic distribution, Mediterranean region was the richest region (226 taxa = 95.8%), followed by Sinai region (172 taxa = 72.9%), Deserts (149 taxa = 63.1%), Nile region (117 taxa = 49.6%), Oases (88 taxa = 37.3%), Red Sea (48 taxa = 20.3%), and Gebel Elba (34 taxa = 14.4%) (Appendix 2). Regarding the chorotype, the Mediterranean region was the most represented (182 taxa), followed by Saharo-Arabian (118 taxa) and Irano-Tauranian regions (75 taxa), while Neotropical(3 taxa) and tropical (2 taxa) regions are less represented. Moreover, eight endemic taxa are recorded.

All the plants in the Mediterranean sand dunes in Egypt offer at least one type of ecosystem services. Medicinal plants were the most represented (187 taxa), followed by grazing (141 taxa), human food (61 taxa), and fuel (47 taxa) categories, while aromatic (9 taxa) and timber (2 taxa) groups were less represented. The life span indicated that annuals were the most represented (115 taxa), followed by perennials (77 taxa), while frutescent (15 taxa) and biennials (5 taxa) were less represented. In addition, twenty-two shrubs and eleven trees were represented. The life form determination showed that therophytes were the most represented (113 taxa), followed by chamaephytes (54 taxa), hemicryptophytes (35 taxa), phanerophytes (17 taxa), Geopytes-helophytes (13 taxa), while geophytes were less represented (5 taxa) (Appendix 2). The IUCN assessment of these taxa showed that the majority of these taxa are still not evaluated (88 taxa), one taxon (*Euphorbia prostrate*) was critically endangered, 2 taxa were endangered, while 44 taxa were least concern and one taxon (*Aegilops bicornis*) was near threatened (Appendix 2).

### Monitoring and prediction of land use/land cover changes

**Fig. 5:**
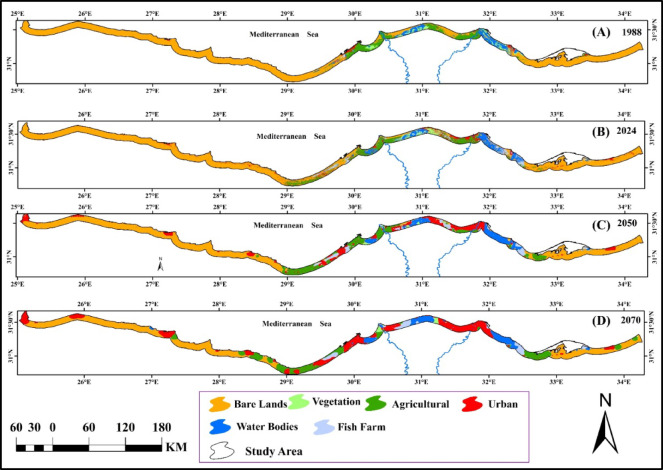
Map represents land cover change between 1988 and 2070 in the whole Mediterranean coastal strip region in Egypt.The colors represent land use type.

**Fig. 6:**
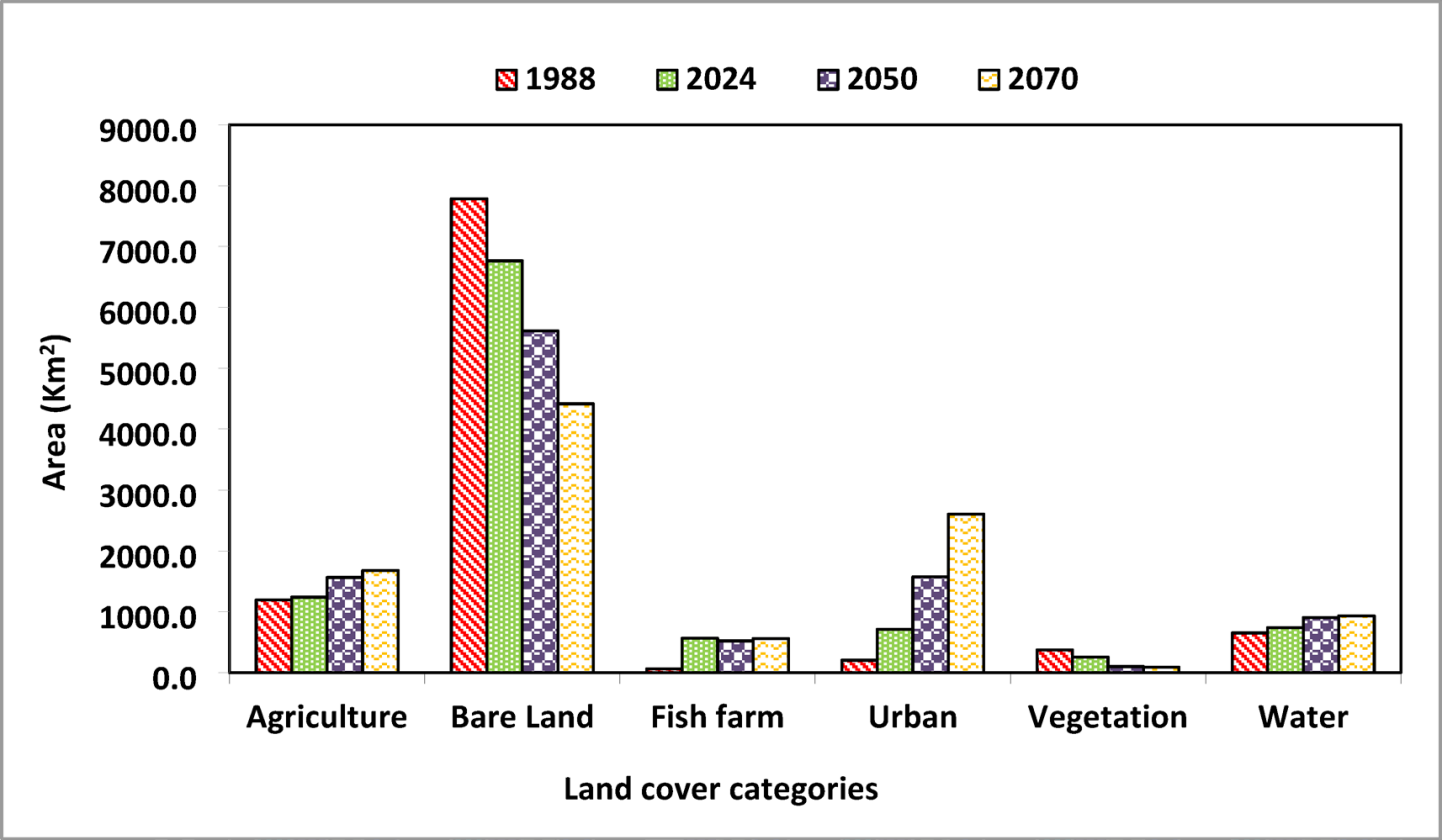
Bar graph showing the distribution area (km^2^) of land cover in the Mediterranean coastal strip region in Egypt.

The supervised land cover classification was conducted using the Google Earth Engine platform, employing a Support Vector Machine (SVM) algorithm on Landsat satellite imagery from 1988, 2024, 2050, and 2070. The approach successfully distinguished spectral and textural features of different land cover classes, with a focus on key changes such as: shifts in agricultural land within the Nile Delta, urban growth in northern Egypt’s expanding cities, and coastal alterations along the Mediterranean. Six distinct land cover categories were delineated: agricultural areas, barren lands, urbanized zones, natural vegetation, water bodies, and aquaculture sites (fish farms), as detailed in Fig. 5 and Appendix 3. The analysis revealed significant shifts in land use patterns within the Mediterranean coastal strip the past (1988) to the present (2024) and the future (2050 and 2070), with the most pronounced transformations occurring in urban expansion, vegetation agriculture and aquaculture development. Human impacts categories (urban, agriculture, fish farms) were increased with time, while the bare areas and natural vegetation were decreased over time (Fig. 6 and Appendix 3). Urbanization was found to be a major factor in land cover change which increased from 203.5 km^2^ (1.98%) in 1988 to 2603.6 km^2^ (25.3%) in 2070. The period from 2050 to 2070 is expected to possess the highest change with 1029.7 km^2^. In addition, agricultural land expanded from 1195.6 km^2^ (11.6%) in 1988 to 1240.4 km^2^ (12.1%) in 2024, followed by a further increase to 1565.1 (15.2%) and 1678.6 km^2^ (16.3%) in 2050 and 2070, respectively (Fig. 6 and Appendix 3).

Driven primarily by human impacts, the area of natural vegetation has undergone a severe and progressive decline, shrinking from 373.8 km² (3.46%) in 1988 to 257.9 km² (2.5%) in 2024, with further projections of 100.1 km² (0.97%) by 2050 and 90 km² (0.88%) by 2070. This widespread loss of vegetative cover has, in turn, led to a corresponding reduction in bare land, which decreased from 7785.3 km² in 1988 to 6763.5 km² in 2024, and is projected to fall to 5612.7 km² and 4413.5 km² by 2050 and 2070, respectively (Fig. 6 and Appendix 3).

**Fig. 7:**
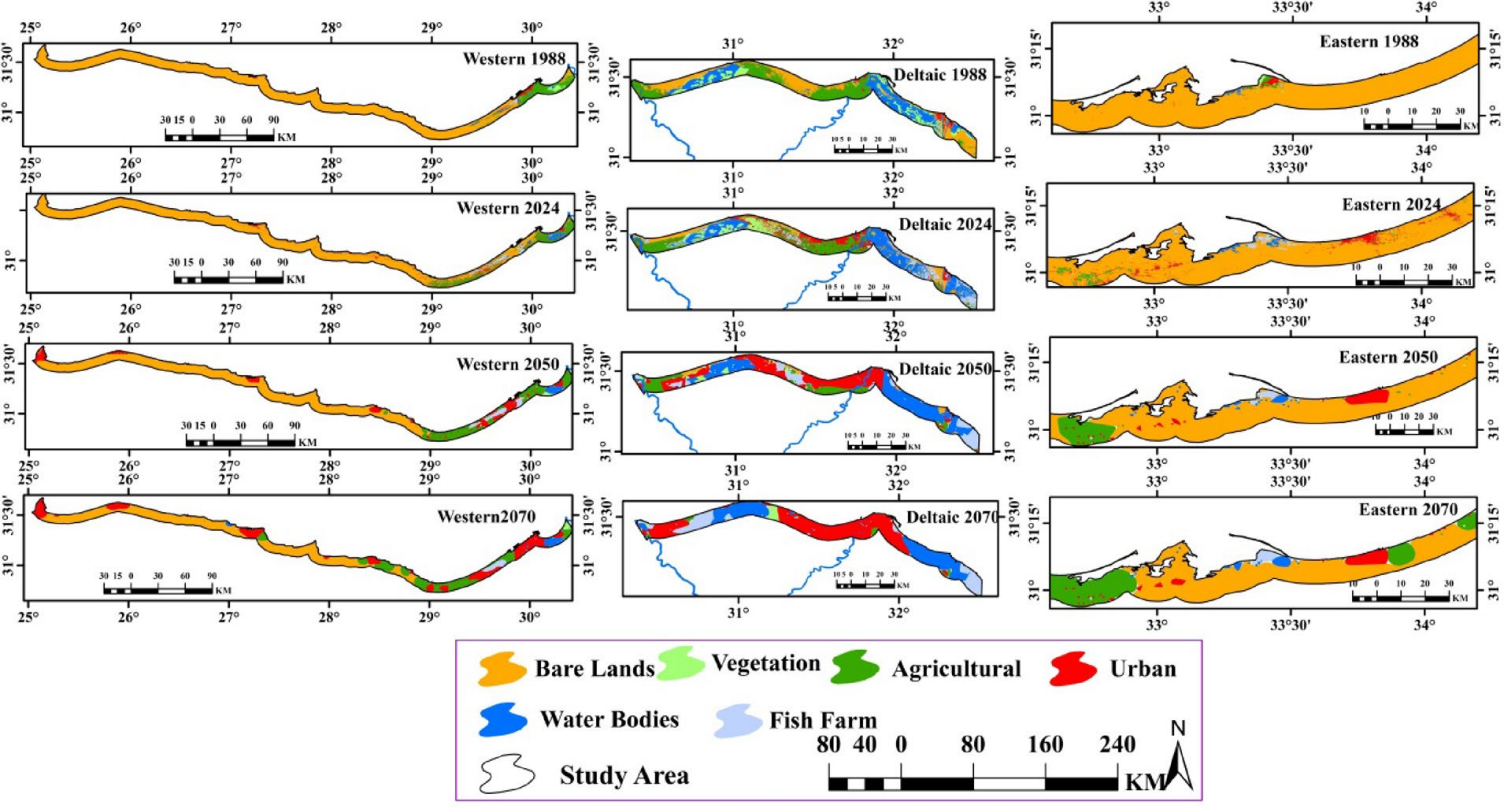
Land cover change between 1988 and 2070 in the three sectors of the Mediterranean coastal strip region in Egypt.

Across Egypt’s three Mediterranean sectors, anthropogenic pressure followed a distinct gradient. The Deltaic sector was the most severely impacted, exhibiting the highest levels of urbanization and the most significant decline in natural vegetation. The Western sector experienced moderate pressure, while the Eastern sector remained the least affected. Consequently, the Eastern sector contained the largest proportion of bare land, followed by the Western sector, with the densely vegetated Deltaic sector containing the least (Fig. 7). The findings provide critical insights into landscape evolution, supporting informed decision-making for sustainable land use and resource management in the region.

### Species distribution modeling

#### Performance of species distribution models

The ensemble model demonstrated strong predictive performance for Egypt’s coastal sand dune vegetation, achieving an AUC score of 0.85 and a TSS score of 0.6, while other evaluation metrics also indicated high model accuracy and reliability (Table 2).

**Table 2:** Accuracy measures used for evaluation of the models predicting the potential distribution of the vegetation of the vegetation of coastal sand dunes habitat in Egypt.

	Threshold	Sensitivity	Specificity	TSS	Kappa	AUC	Overall accuracy
GLM	0.11	0.69	0.77	0.47	0.24	0.8	0.77
BRT	0.06	0.85	0.76	0.61	0.27	0.86	0.77
RF	0.09	0.86	0.81	0.67	0.34	0.89	0.81
SVM	0.07	0.76	0.82	0.58	0.31	0.81	0.82
Maxent	0.38	0.77	0.81	0.58	0.3	0.85	0.81
Ensemble	0.14	0.78	0.79	0.58	0.29	0.84	0.79

**Fig. 8:**
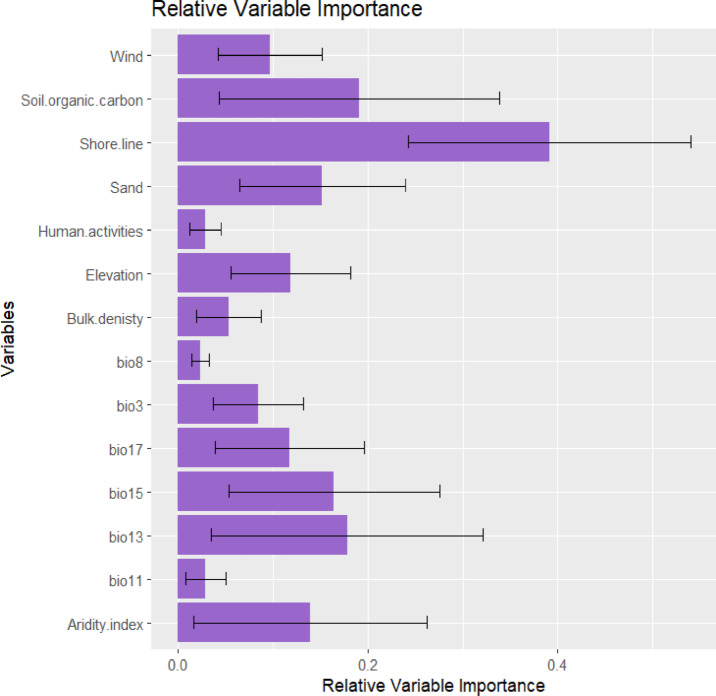
The typical mean proportion of relative significance assigned to environmental factors in ensemble models forecasting the potential distribution of the vegetation in Mediterranean Coastal sand dunes habitat in Egypt.

Fourteen predictor variables were chosen to develop SDMs for the vegetation in the coastal sand dunes habitat based on the results of variance inflation factors (Table 3). The relative importance of the predictor variables contributing to the ensemble model revealed that distance from shore line, soil organic carbon, Bio13, Bio15, and sand were the most important variables controlling the distribution of vegetation in the sand dunes habitat, with mean contributing variables equal to 39.1, 19.1, 17.9, 16.5, and 15.2%, respectively (Fig. 8).

**Table 3:** Summary of the selected environmental predictor variables that account for the possible distribution of vegetation of the mediterranean coastal sand dunes habitat. VIF: variance inflation factor.

Code	Variable	VIF
Bio 3	Isothermality (Bio 2/Bio 7) × 100	2.2
Bio 8	Mean temperature of wettest quarter	3.3
Bio 11	Mean temperature of coldest quarter	2.1
Bio 13	Precipitation of wettest month	3.1
Bio 15	Precipitation seasonality	3.4
Bio 17	Precipitation of driest quarter	1.4
Bulk density		4.8
Soil organic carbon		3.9
Sand		2.8
Elev (m)	Elevation	2.1
Human activities		1.9
shore line	Distance from shore line	2.0
Wind speed		2.5
Aridity index		1.2

**Fig. 9:**
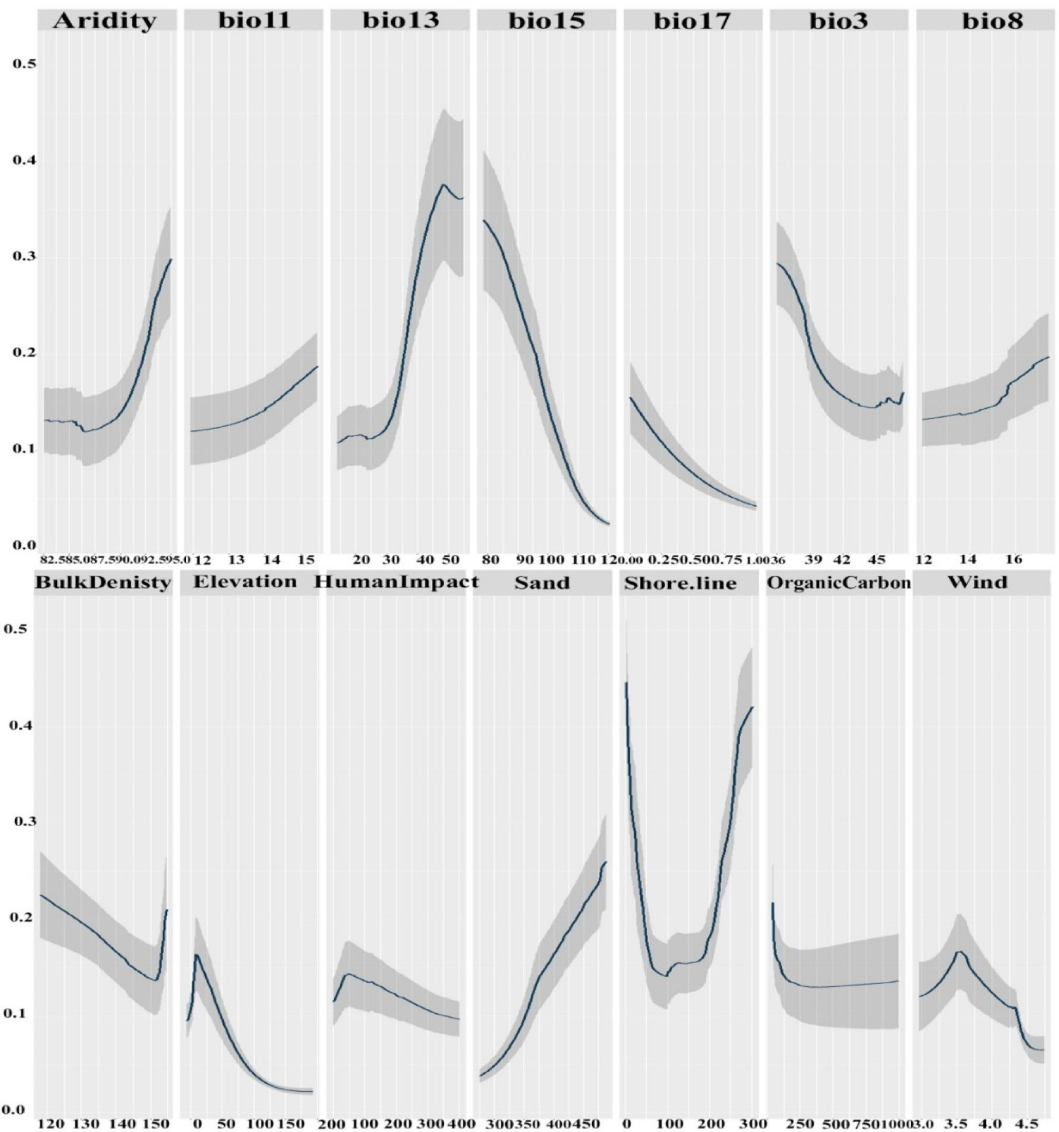
The predictor variables’ response curves were utilized in the distribution modeling of the vegetation in Mediterranean Coastal sand dunes habitat in Egypt.

The response curves revealed the influence of predictive variables on habitat suitability (Fig. 9). The probability of occurrence showed a positive correlation with Bio 8 (mean wet quarter temperature), Bio 11 (mean cold quarter temperature), Bio 13 (precipitation of the wettest month), and sand content. In contrast, it was negatively correlated with Bio 3 (isothermality), Bio 15 (precipitation seasonality), Bio 17 (precipitation of the driest quarter), human activity, and soil organic carbon. Furthermore, the relationship was unimodal (hump-shaped) for elevation and wind speed, where probability increased to an optimum before declining. Conversely, the relationship was trough-shaped for bulk density and distance from the shore, with probability initially decreasing before rising again beyond a certain threshold.

#### Current and future predictions

**Fig. 10:**
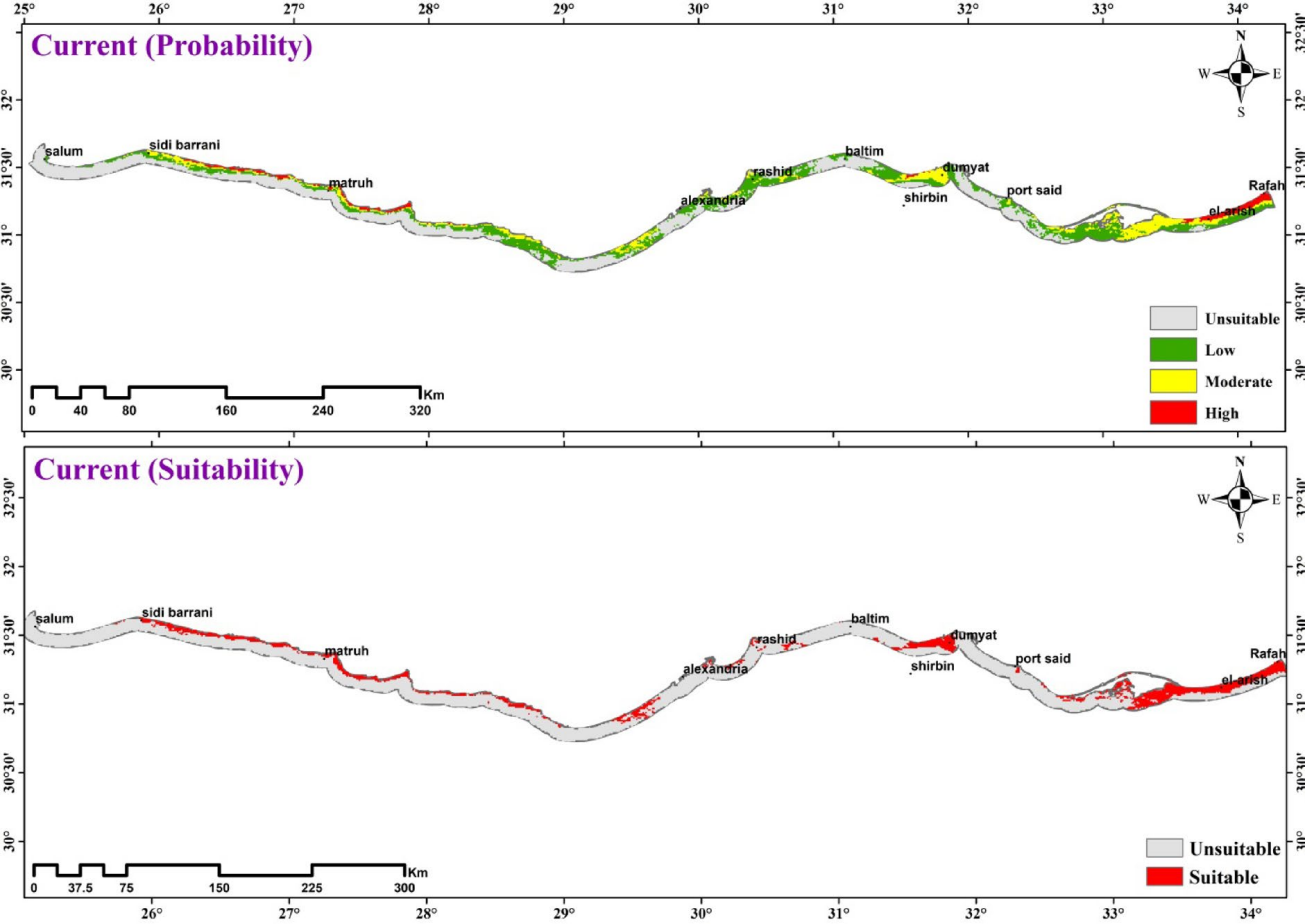
The current climate suitability map for the vegetation in the Mediterranean Coastal sand dunes habitat in Egypt.

Based on ensemble model projections, the total potentially suitable area for Mediterranean coastal sand dune vegetation in Egypt under current climate conditions is 1911 km², covering 19.52% of the total study area (Fig. 10; Table 4). Vegetation coverage is most extensive in the Eastern and Western Mediterranean sectors, while being scarce in the Deltaic Mediterranean region. The suitability classes are distributed as follows: 1559 km² (low suitability), 278 km² (moderate suitability), and 74 km² (high suitability). The most favorable habitats are located in Rafah, Mattala, Hosinat, El-sheikh Zewaid, El-Arish, and Ahtam in North Sinai, with smaller suitable areas identified in Gamasa, Ras El-Hekma, and Matroun in the Western Mediterranean.

**Table 4:** Comparison between current and future habitat suitability of the vegetation in the mediterranean coastal sand dunes habitat in Egypt.

Scenario	Distribution suitability		Habitat suitability			
	Unsuitable	Suitable	Loss	Unsuitable	Stable	Sain
Current	7877	1911				
SSP126 2041–2060	8197	1591	1415	6782	441	1150
SSP126 2061–2080	8101	1687	1472	6629	384	1303
SSP585 2041–2060	8106	1682	1294	6812	562	1120
SSP585 2061–2080	8049	1739	1234	6815	622	1117

**Fig. 11:**
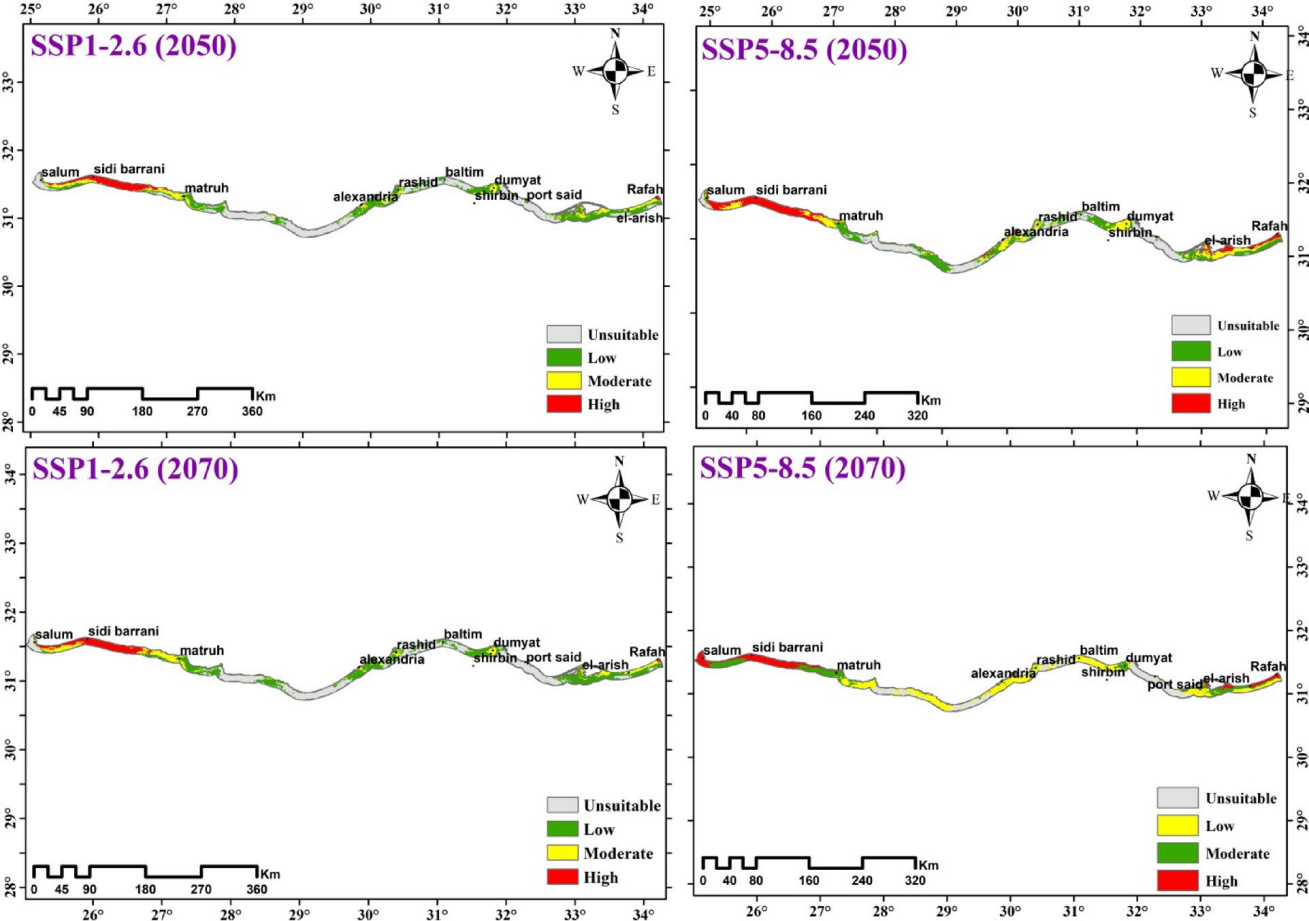
Predicted distribution of the vegetation in the Mediterranean coastal sand in Egypt under two SSP1-2.6 and SSP5-8.5 during the period 2041–2060 and 2061–2080.

The future distribution of the vegetation in the Mediterranean Coastal sand dunes habitat in Egypt, as predicted by the ensemble model under SSP1-2.6 and SSP5-8.5 scenarios using IPSL-CM6A model, is shown in Fig. 11 and Table 4. The potential future changes in habitat suitability of Mediterranean Coastal sand dunes vegetation were slightly similar under both climate change scenarios. The two scenarios revealed a decline in the vegetation in the sand dunes habitat, with higher decline percentage in the period 2041–2060 than 2061–2080. In addition, SSP1-2.6 scenario showed higher decline percentage than SSP5-8.5 scenario in the two periods.

Projections for 2041–2060 under the SSP1-2.6 scenario (IPSL-CM6A-LR model) indicate a decline in suitable habitat for Mediterranean coastal sand dune vegetation in Egypt to approximately 1591 km² (16.25% of the total study area). By 2070 under the same scenario, the suitable area is projected to recover slightly to 1687 km² (17.24%). Spatial analysis reveals that high vegetation richness remains concentrated in the western Mediterranean dunes, particularly from Matrouh to Sidi Barrani (encompassing Zoairat, Zewida, Abu Marzouk, and Sidi Barrani), and in the Rafah region of the eastern sector. Areas of medium occurrence are primarily found in El-Sheikh Zewaid and El-Arish (eastern sector), Damietta and Rashid (Deltaic sector), and Matrouh and parts of Sidi-Barrani (western sector). Conversely, low vegetation occurrence dominates the western dunes of Ras El-Hekma and Sidi-Abdelraham, most of the Deltaic sector, and the eastern areas of Bear El-Abd, El-Ahtam, and El-Taira (Fig. 11; Table 4).

#### Potential habitat change of the vegetation in the Mediterranean Coastal sand dunes habitat in Egypt at different climates

**Fig. 12:**
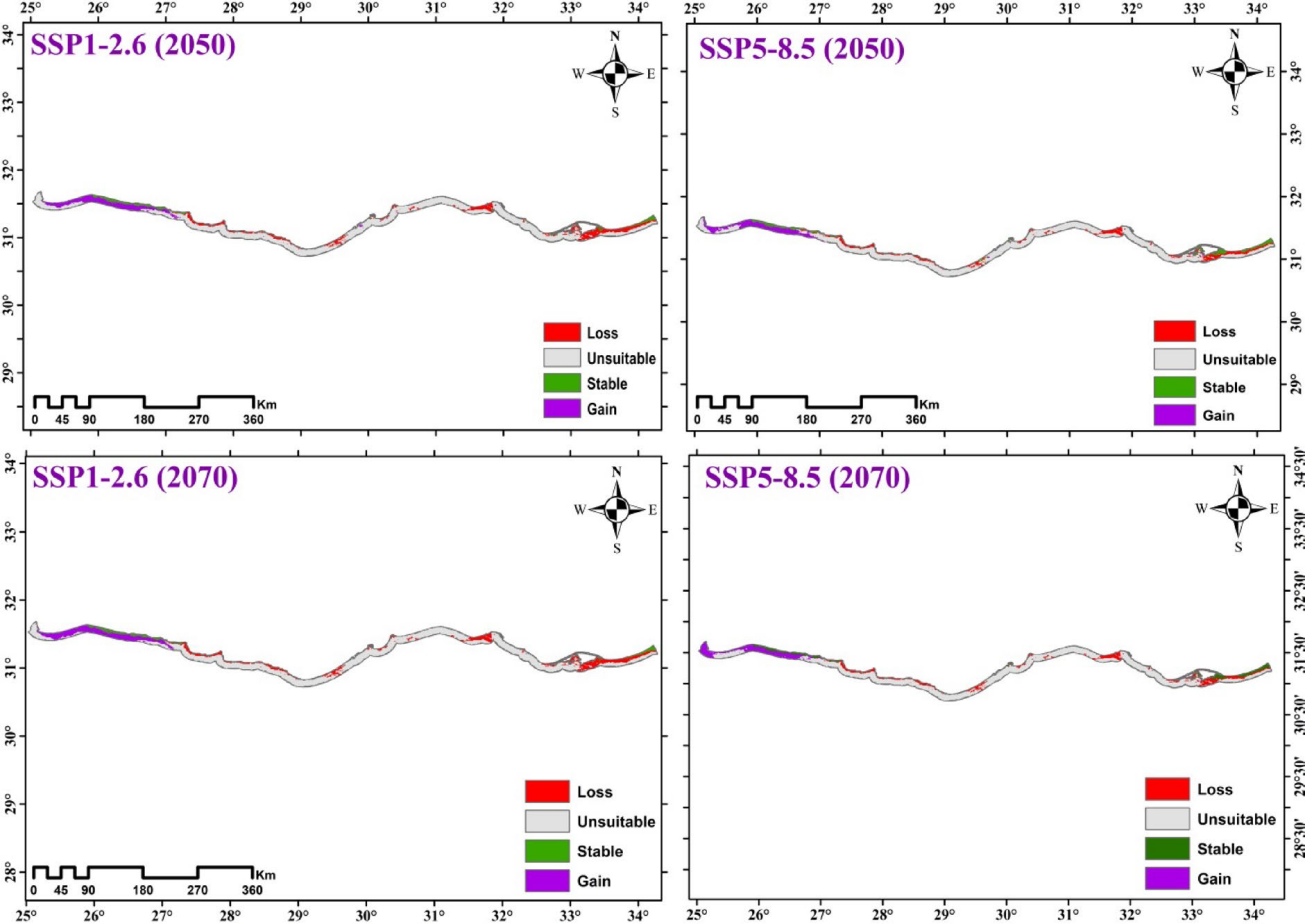
The habitat suitability map of the vegetation in the Mediterranean coastal sand dunes in Egypt under two SSP1-2.6 and SSP5-8.5 during the period 2041–2060 and 2061–2080.

The ensemble model predicts a decline in the distribution of the vegetation in the Mediterranean coastal sand dunes in Egypt under different climate change scenarios (SSP1-2.6 and SSP5-8.5) for the near (2041–2060) and far future (2061–2080) compared with its current range. According to the IPSL-CM6A-LR model, the species’ suitable habitat will persist in small regions of Rafah, El-Arish and El-Sheikh Zewaid in the Eastern Mediterranean, and small regions from Matrouh to Siddi Barrani in the Western Mediterranean. The majority of loss areas were concentrated in the Western Mediterranean from Alexandria to Sidi Barrani, Damietta and Gamasa in the Deltaic Mediterranean, El- Rouda, El-Karouba and El-Ssheikh Zewaid in the Eastern Mediterranean. On the other hand, the gain areas were concentrated in the Western Mediterranean from Matrouh to El-Sallum (Fig. 12 and Table 4).

By 2050, model projections indicate a habitat loss (sand dunes area) of 1415 km² under SSP1-2.6 and 1294 km² under SSP5-8.5. This loss is counterbalanced by significant expansion in western regions, projected at 1150 km² and 1120 km² for the respective scenarios. A similar pattern is projected for 2070, with a net increase in total suitable area compared to 2050. While habitat loss is expected to persist (1172 km² under SSP1-2.6 and 1234 km² under SSP5-8.5), western expansion is projected to be substantial, reaching 1303 km² and 1117 km². Overall, these results forecast a major westward range shift for the dune vegetation. The more pronounced expansion under SSP1-2.6 by 2070 suggests that the higher-emission scenario (SSP5-8.5) may ultimately be less favorable for sustained habitat gains despite the initial lower rate of loss (Fig. 12 and Table 4).

## Discussion

Egypt’s northern Mediterranean coast, part of a globally significant biodiversity hotspot, supports diverse plant communities shaped by variations in soil, terrain, and climate. Seasonal rains trigger the growth of annual plants, while prolonged dry periods favor resilient woody shrubs and perennial herbs, resulting in sparse scrub vegetation with occasional dense patches^8,107^. Despite covering just 1.6% of Earth’s land area, this region harbors 10% of the world’s higher plant species, underscoring its ecological importance^108^. Most of the sand dunes close to the Mediterranean coast of Egypt have been destroyed due to human pressures^53^. Batanouny^12^ pioneered the recognition of Egypt’s Mediterranean coastal dunes as a threatened ecosystem. Subsequent studies have documented extensive habitat modification in these areas. Given these environmental pressures, evaluating the conservation status of dune vegetation communities becomes essential to safeguard these fragile ecosystems from further degradation or irreversible habitat destruction^14,21,53^.

### Checklist and floristic analysis

The present study indicated that the Mediterranean coastal sand dunes in Egypt include 236 taxa belonging to 75 genera and 39 families. The majority of the previous studies have been conducted in the western Mediterranean region. For instance, Shaltout and Ahmed^20^ recorded 548 taxa in the different habitats including coastal sand dunes of the Western Mediterranean region, Abdelaal^53^ recorded 113 species in the Western Mediterranean sand dunes, Hamed^16^ recorded 116 taxa in the Mediterranean coastal sand dunes from Alexandria westward to Mersa Matruh, while Arief^14^ recorded 237 species distributed in 13 different habitats, with 19 species inhabit dune only in the Western Mediterranean region in Egypt. Multiple studies have documented a reduction in species diversity, likely associated with human-induced habitat disruption. This trend mirrors observations across Mediterranean coastal regions, where anthropogenic activities have been identified as the primary driver of biodiversity loss.

Among the recorded taxa in the Mediterranean coastal sand dunes in Egypt, 8 taxa are endemic which were assessed by El-Khalafy^109^ according to IUCN categories. Six taxa were assessed as threatened, while two (*Bellevalia salaheidii* and *Muscari albiflora*) were believed to be extinct. These taxa exhibit a highly restricted distribution with limited population numbers, facing multiple threats to their survival^110,111^. Their populations are severely fragmented, with evident deterioration in habitat quality. Primary threats include climate change impacts, particularly extreme temperatures and prolonged drought conditions. In addition, these taxa also face significant pressure from human activities, including construction and land development. Local Bedouin agricultural practices near its habitat have contributed to habitat clearance; while tourism, overgrazing, and intensive scientific collection have further disturbed populations^109^.

Also, these taxa have suffered particularly from shoreline development. The establishment of tourist resorts has resulted in both direct mortality of mature plants and widespread habitat destruction. Infrastructure projects, particularly road construction through dune systems (e.g., in Matrouh), have significantly disrupted their habitat^21,109^. Mediterranean Delta regions have experienced especially severe impacts, where developments like Marina Delta lagoons and coastal gas stations have eliminated extensive dune habitats supporting vulnerable species such as *Echinops taeckholmianus* and *Pancratium arabicum*. Additional large-scale projects including Delta University, New Mansoura University, New Mansoura City, and the Gamasa industrial complex along the Baltim-Gamasa coastline have caused substantial degradation of remaining natural ecosystems^112^.

### Remote sensing and species distribution modeling

#### Model performance and variable importance

The spatial distribution of species is governed by multiple interacting factors spanning physical, chemical, and biological parameters^113^. Key edaphic variables including soil thermal regimes, hydric conditions, and nutrient dynamics exert significant control over organismal distributions across landscapes^114^. This study identified distance from shore, soil organic carbon, Bio13, Bio15, and sand content as the key factors governing dune vegetation distribution. Habitat suitability increased with higher Bio8, Bio11, Bio13, and sand content, but decreased with Bio3, Bio15, Bio17, elevation, human disturbance, and soil organic carbon. Notably, elevation and wind speed exhibited hump-shaped relationships with suitability, while bulk density and distance from shore showed U-shaped relationships.

In Coastal Mediterranean sand dunes, vegetation distribution is primarily controlled by a strong sea-inland environmental gradient, where factors like soil properties, salt spray, and sand mobility decrease landwards, creating distinct plant zones from pioneer annuals on the fore-dune to shrubs inland^115,116^. However, this natural zonation is increasingly altered by anthropogenic pressures; tourism and urbanization reduce typical, habitat-structuring psammophytes and facilitate their replacement by nitrophilous ruderal species, which do not perform the same dune-stabilizing functions^115,117^. Furthermore, coastal erosion, often exacerbated by human activities, acts as a significant indirect driver of plant diversity loss by altering the landscape configuration upon which these specialized plant communities depend^115^.

Our results are partially agreed with^118^, who showed remote sensed data contribution in predicting the distribution of three native Mediterranean plants inhabiting sand dunes habitat. The distance to coastline, Bio8, Bio9, Bio1, Bio18 and Bio19 were the most contributed variables in the distribution of *Thymelea hirsuta*, *Ononis vaginalis* and *Limoniastrum monopetalum*. Recent research has identified key environmental drivers shaping coastal vegetation patterns across different Mediterranean regions. Halmy^119^ demonstrated that elevation, climatic parameters, and coastal proximity significantly influence the distribution of important plant species in Egypt’s northern coastal deserts. These findings align with earlier foundational work by Guara-Requena^120,121^ in Spanish coastal ecosystems, which established topography, distance from shoreline, and habitat alteration as the three fundamental factors governing the spatial distribution of sand-loving (psammophilous) plants and dune plant communities.

Climatic factors governing annual cycles (particularly temperature and precipitation regimes) appear to significantly influence total vegetation coverage. These parameters create a stark ecological contrast between humid coastal zones (mediated by Mediterranean and Atlantic maritime influences) and arid inland regions, as demonstrated by Walas and Taib^122^. The current study’s results are supported by Halmy^123^ and Abdelaal^124^ that demonstrate that seasonal climatic patterns particularly influence species distribution at local scales. Specifically, Abdelaal^124^ identified mean temperature of the wettest quarter (Bio 8) and precipitation seasonality (Bio 15) as crucial determinants of species richness in Egypt’s Mareotis subsector. These observations partially align with earlier findings by Zahran^125^, Zahran and Willis^8^, and Frihy and El-Sayed^126^ that confirming the consistent importance of these bioclimatic factors across different studies.

Distance from the shoreline creates a strong environmental gradient of salt spray exposure, wind intensity, and soil salinity. In addition, it controls species zonation from pioneer beach species to more stable inland communities^127^. Soil organic carbon governs nutrient availability in inherently nutrient-poor dune soils and enhances water retention capacity in porous sandy substrates^128^. Moreover, Precipitation of wettest month (Bio 13) determines critical water availability for germination and establishment and influences seasonal growth patterns of dune specialists^129^. In addition, Precipitation seasonality (Bio 15) affects plant stress tolerance and phenological adaptations and shapes competitive interactions between annuals and perennials^15^. Sand content determines substrate mobility and erosion potential and influences root architecture and water infiltration rates^130^.

#### Current and future predictions

Ecological niche modeling and species distribution models (SDMs) have become fundamental tools in climate change biology^131^, enabling researchers to evaluate how shifting climatic conditions may alter species distributions and geographic ranges^131,132^. These approaches gain particular predictive power when combining climate model projections with land-use change data to assess cumulative impacts on species distributions^132^. Advances in General Circulation Models (GCMs) and refined climate change scenarios now permit more accurate projections of distributional shifts^133^. However, proper model evaluation and recognition of methodological limitations remain crucial to prevent misinterpretation of results and ensure sound conservation planning^134^. Species responses to climate change vary significantly based on their physiological and phenological adaptations^135^, with ecological impacts already manifesting as measurable range shifts and distributional changes across numerous taxa^136^.

The current analysis reveals distinct regional patterns in dune vegetation distribution, with significantly higher vegetation percentage observed in both Eastern and Western Mediterranean coastal dunes compared to the notably sparse vegetation cover characteristic of Deltaic Mediterranean sand dune systems. The richness of the Eastern and Western Mediterranean coastal dunes with the vegetation due to that Western (e.g., Matrouh) and Eastern (e.g., Sinai) dunes exhibit more stabilized geomorphology with older sand formations, allowing vegetation succession. In contrast, the Deltaic coast experiences frequent sediment redistribution from Nile discharges, preventing plant establishment^137^. In addition, Nile Delta has experienced 94% of Egypt’s coastal urbanization and intensive agriculture that reducing native dune area^138^. Between 1998 and 2014, the Nile Delta’s coastal stretch from Rashid to Damietta experienced substantial urban growth, with a net expansion of approximately 35,900 ha, peaking during the 2011–2013 period^34,139,140^. This rapid urbanization has driven profound land use/land cover (LULC) transformations, creating measurable climatic impacts across the region^22,23,141^. The conversion of natural landscapes to build environments has replaced permeable vegetated surfaces with heat-retentive impervious materials (e.g., asphalt, concrete), fundamentally altering local surface energy balances through increased heat absorption and reradiation^142,143^ .

The ensemble modeling projections for IPSL-CM6A-LR climate scenarios indicated consistent trends in habitat suitability changes for the vegetation in the Mediterranean coastal sand dunes in Egypt under SSPs126 and SSPs585 pathways. Results predict a decline in the vegetation of the Mediterranean coastal sand dunes in Egypt. Our results agreed with research by Fenu^144^, Fenu^116^, Ciccarelli^15^, Abdelaal^53^, and Arief^137^ that similarly reported declining plant species richness in Egypt’s deltaic Mediterranean coastal dunes, reinforcing the widespread impact of human disturbance on these ecosystems. The decline areas in 2050 were slightly larger than that of 2070. The analysis revealed distinct spatial patterns in vegetation changes across Egypt’s Mediterranean coast, with significant losses concentrated in three key regions: 1- western sector between Alexandria and Sidi Barrani, 2- Deltaic zone encompassing Damietta and Gamasa, and 3- eastern areas of El-Rouda, El-Karouba, and El-Sheikh Zewaid. Conversely, vegetation gains were predominantly localized in the far western coastal stretch extending from Matrouh to El-Sallum, suggesting more favorable ecological conditions or reduced anthropogenic pressures in this particular region compared with other Mediterranean coastal areas. A study in central Italy demonstrated that remote sensing could effectively model the invasion of alien plant species (e.g., *Carpobrotus spp.* and *Agave americana*) in coastal dune ecosystems by quantifying proxies for propagule pressure, abiotic conditions, and biotic interactions, thereby identifying areas most vulnerable to future invasions. The comparison of data between 2003 and 2017 revealed a substantial decline in the cover of psammophytes^117^.

The Mediterranean region is a key hub of plant biodiversity and a global hotspot for species richness^145,146^. However, it faces significant threats from both human activities and natural pressures, leading to widespread fragmentation of natural habitats. Over the last four millennia, this area has been home to some of history’s most influential civilizations, resulting in intensive land use and the transformation of much of its native vegetation into farmlands^147^. While topographic and soil conditions interact with and are often modified by human activities plant species^104^, particularly those endemic to Mediterranean sand dunes, demonstrate significantly greater vulnerability to anthropogenic disturbances compared with widespread generalist species^104,148–150^ .

Furthermore, numerous species face threats from excessive harvesting and cutting for commercial trade, driven by local communities and herbalists seeking medicinal plants. This practice is particularly harmful as it disproportionately affects rare and geographically restricted species, pushing them further toward decline. Meanwhile, the growing demand for fuel wood among Bedouin populations is putting additional pressure on larger woody perennials, particularly those with dense branches and deep root systems. The loss of these key woody species often marks the beginning of broader ecological disruption, ultimately leading to the irreversible transformation of natural landscapes^17^.

Numerous studies have examined the floristic diversity of the Mediterranean coastal region, highlighting the significant impact of human-induced habitat destruction, particularly urbanization^5,21,109,148^. Others have concentrated on specific environmental influences, such as soil factors^53,144^. However, endangered habitats face severe threats not only from urbanization but also from the unsustainable exploitation of ecosystem services.

For instance^151^, noted that Egypt’s fragile northwestern coastal desert, despite its low productivity, supplies various essential goods and services, putting these habitats at risk of degradation and potential species extinction. Similarly, the Nile Delta is experiencing accelerating habitat loss due to unregulated human use of natural flora without proper monitoring or conservation measures^152^. Comparable challenges have been observed in the Gulf of Gabès in southern Tunisia, where disturbance-driven biodiversity decline has been documented^153^. Moreover, Urbanization has been identified as the most destructive factor affecting coastal plant communities, causing severe habitat destruction and pushing many species toward extinction. This widespread habitat degradation consequently deprives ecosystems of their vital services^17,151,154^.

In addition, Egypt has experienced significant temperature increases, likely exacerbated by widespread deforestation, which has compounded natural habitat degradation. The^155^ projected temperature rises of 0.2 °C per decade, with potential increases of 1.5–3.5 °C by century’s end depending on emission scenarios. While these predictions accounted for fossil fuel emissions and forests’ carbon sequestration capacity, Egypt’s continued tree loss has tragically accelerated these climate impacts.

The region’s agricultural roots date back to Greco-Roman times, when olive and grape cultivation thrived. However, climatic shifts and the drying of the Nile’s eastern distributaries forced a transition to pastoralism and dryland farming, which persisted into modern times. Recent projections indicate significant cropland expansion, quarrying, and coastal resort development, driven by Egypt’s strategy to reclaim desert areas for agriculture and tourism^119^. These changes (particularly irrigation infrastructure and dune resort construction) have degraded natural vegetation, pressuring rangelands, salt marshes, and peri-urban zones. Such landscape modifications threaten native biodiversity, with habitat loss and fragmentation expected to shrink ecological niches and reduce species populations^151^.

## Conclusion

This study reveals that climate change and anthropogenic effects threatens Egypt’s vegetation in the Mediterranean coastal sand dunes in Egypt, with unchecked urbanization accelerating habitat degradation and risking flora extinction. Using remote sensing and SDM, we confirmed these plants effectively indicate ecosystem health and conservation status. The findings highlight the urgent need to protect Mediterranean coastal sand dunes from destructive human activities through enforced conservation measures.

## Supplementary Information

Below is the link to the electronic supplementary material.

Supplementary Material 1

## Data Availability

The datasets used and/or analyzed during the current study are available from the corresponding author on reasonable request.
