# Supplementary material for: Investigating sand dunes flora conservation based on remote sensing and predictive modeling in the Mediterranean coastal region, Egypt
Source: Sci Rep. 2025 Dec 10;15:43583. doi: 10.1038/s41598-025-29710-y (PMC12698863; doi:10.1038/s41598-025-29710-y)
Supplement: Supplementary file 1 — Supplementary Material 1 [file 41598_2025_29710_MOESM1_ESM.docx]

**Appendix 1** Environmental variables used in the study

| **Variable** | **Code** | | **Source** | | **units** |
| --- | --- | --- | --- | --- | --- |
| **Climatic/Bioclimatic variables** | | | | | |
| **Annual mean temperature** | Bio1 | WorldClim | | | °C |
| **Mean diurnal range (max. Temp- min. temp)** | Bio | WorldClim | | | °C |
| **Isothermality (Bio2/Bio7) × 100** | Bio3 | WorldClim | | | °C |
| **Temperature seasonality (SD × 100)** | Bio4 | WorldClim | | | °C |
| **Max temperature of warmest month** | Bio5 | WorldClim | | | °C |
| **Min temperature of coldest month** | Bio6 | WorldClim | | | °C |
| **Temperature annual range (Bio5-Bio6)** | Bio7 | WorldClim | | | °C |
| **Mean temperature of wettest quarter** | Bio8 | WorldClim | | | °C |
| **Mean temperature of driest quarter** | Bio9 | WorldClim | | | °C |
| **Mean temperature of warmest quarter** | Bio10 | WorldClim | | | °C |
| **Mean temperature of coldest quarter** | Bio11 | WorldClim | | | °C |
| **Annual precipitation** | Bio12 | WorldClim | | | mm |
| **Precipitation of wettest month** | Bio13 | WorldClim | | | mm |
| **Precipitation of driest month** | Bio14 | WorldClim | | | mm |
| **Precipitation seasonality** | Bio15 | WorldClim | | | mm |
| **Precipitation of wettest quarter** | Bio16 | WorldClim | | | mm |
| **Precipitation of driest quarter** | Bio17 | WorldClim | | | mm |
| **Precipitation of warmest quarter** | Bio18 | WorldClim | | | mm |
| **Precipitation of coldest quarter** | Bio19 | WorldClim | | | mm |
| **minimum temperature** | tmin | WorldClim | | | °C |
| **maximum temperature** | tmax | WorldClim | | | °C |
| **average temperature** | tavg | WorldClim | | | °C |
| **Precipitation** | Prec | WorldClim | | | mm |
| **wind speed** | Wind | WorldClim | | | m s^-1^ |
| **water vapor pressure** | Vapr | WorldClim | | | kPa |
| **Climatic moisture content** |  | ENVIREM | | | - |
| **Solar radiation** | Srad |  | | | kJ m^-2^ day^-1^ |
| **Degree of water deficit below water need** | Aridity index | ENVIREM | | | - |
| **Topographic variables** | | | | | |
| **Elevation** | Elev (m) | https://www.usgs.gov | | m | |
| **Slope** | SL (%) | derived from elevation | | (%) | |
| **Aspect** | AS (degrees) | derived from elevation | | degree | |
| **Soil factors** | | | | | |
| **Organic carbon density** |  | Soilgrid | | g/dm^3^ | |
| **Soil organic carbon stock** |  | Soilgrid | | g/m² | |
| **Bulk Density** |  | Soilgrid | | cg/cm^3^ | |
| **Caly content** |  | Soilgrid | | g/kg | |
| **Coarse fragment** |  | Soilgrid | | cm^3^/dm^3^ | |
| **Sand** |  | Soilgrid | | g/kg | |
| **Silt** |  | Soilgrid | | g/kg | |
| **Cation exchange capacity** |  | Soilgrid | | mmol(c)/kg | |
| **Nitrogen** |  | Soilgrid | | cg/kg | |
| **Soil organic carbon** |  | Soilgrid | | g/kg | |
| **pH water** |  | Soilgrid | | pH × 10 | |
| **Vol. water content at -10kpa** | Water10 | Soilgrid | | m³/m³ | |
| **Vol. water content at -33kpa** | Water33 | Soilgrid | | m³/m³ | |
| **Vol. water content at -1500kpa** | Water1500 | Soilgrid | | m³/m³ | |
| **Anthropogenic effects** | | | | | |
| **Human footprint** | Human impacts | https://hub.arcgis.com | |  | |

**Appendix 2** Checklist of the plants in the coastal Mediterranean sand dunes in Egypt. The phytogeographical regions are abbreviated as follows: N: Nile region, O: Oases of the western desert, M: Mediterranean coastal region, D: All deserts of Egypt except that of Sinai, De: Desert east of the Nile, Dw: Desert west of the Nile, R: Red Sea region, GE: Gebel Elba region and S: Sinai Peninsula (After Boulos, 2009). Chorotypes are coded as follows: ME: Mediterranean, IT: Irano-Turanian, SA: Saharo-Arabian, ES: Euro-Siberian, SZ: Sudano-Zambezian. Economic importance are coded as follows: Gr: grazing, Md: medicinal, Hf: Human food, Fu: fuel wood, Ar: Aromatic, Orn: ornamental, Ot, others.The life span is coded as follows: Per.: Perennial, Ann.: Annual, Frut.: Frutescent. The life forms are coded as follows: GH; geophyte-helophyte, GE: geophyte, TH: therophyte, CH: chaemaphyte, PH: phanerophyte and HE: hemicryptophyte. The conservation categories are coded as follows: EN: endangered, VU: vulnerable, LC: least concern, NE: Not evaluated, CR: critically endangered, EX: extinct and DD: data deficient. * referes to plants that restricted to sand dunes habitat.

| Species | Family | Local distribution | Chorotype | Economic importance | Life span | Life form | IUCN status |
| --- | --- | --- | --- | --- | --- | --- | --- |
| *Acacia saligna* (Labill.) H.L.Wendl. | Fabaceae | M | PAN | Gr, Fu | Tree | PH | LC |
| *Achillea fragrantissima* (Forssk.) Sch.Bip. | Asteraceae | M, D, R, S | ME+ SA+ SZ | Gr, Md, Ar, Fu | Frut. | HE | NE |
| *Achillea maritima* (L.) Ehrend. & Y.P.Guo | Asteraceae | M, S | ME+ ES | Md | Frut. | HE | NE |
| **Achillea tenuifolia* Lam. | Asteraceae | O, M, D, S | SA+ME | Gr, Md, Ar, Fu | Per. | HE | NE |
| *Adonis dentata Delile* | Ranunculaceae | M, D, S | SA+ IT+ ME | Md | Ann. | TH | NE |
| *Aegilops bicornis* (Forssk.) Jaub. & Spach | Poaceae | N, M, S | ME | Gr, Md, Fu | Ann. | TH | NT |
| *Aegilops kotschyi* Boiss. | Poaceae | O, M, S | IT+ SA | Gr, Md, Fu | Ann. | TH | LC |
| *Aeluropus lagopoides*(L.) Thwaites | Poaceae | N, O, M, D, R, GE, S | ME+ IT+ SA+ SZ | Gr, Ed, Fu | Per. | GH | LC |
| *Alhagi graecorum* Boiss. | Fabaceae | N, O, M, D, R, S | ME+IT | Gr, Md,Hf, Fu | Frut. | HE | NE |
| *Allium aschersonianum* Barbey | Amaryllidaceae | M | IT | Me, Hf | Per. | GE | NE |
| *Allium erdelii* Zucc. | Amaryllidaceae | M, S | ME+IT | Me, Hf | Per. | GH | NE |
| *Allium mareoticum* Bornm. & Gauba | Amaryllidaceae | M | Endemic | Hf, Me | Per. | GH | NE |
| *Allium roseum* L. var. *tourneauxii* Boiss. | Amaryllidaceae | M | ME | Gr, Md, Hf | Per. | GH | NE |
| *Ammi majus* L. | Apiaceae | N, O, M, S | ME+IT | Md, Hf, Ot | Ann. | TH | NE |
| *Anabasis articulata* (Forssk.) Moq. | Amaranthaceae | O, M, D, S | SA+ME | Fu | Shrub | CH | NE |
| *Anacyclus monanthos* (L.) Thell. subsp. *monanthos* | Asteraceae | M, D | ME+SA | Gr, Fu | Ann. | TH | NE |
| *Anchusa aegyptiaca* (L.) A.DC. | Asteraceae | N, M, D, S | ME+IT | Gr | Ann. | TH | NE |
| *Anthemis microsperma* Boiss & Kotschy | Asteraceae | M, S | Endemic | Md, Gr | Ann. | TH | NE |
| *Arisarum vulgare* O.Targ.Tozz. | Araceae | M, S | ME | Gr, Md, Hf | Per. | GE | NE |
| *Arnebia decumbens* (Vent.) Coss. & Kralik | Boraginaceae | D, M., S. | SA+ME | Md | Ann. | TH | NE |
| *Artemisia herba-alba* Asso | Asteraceae | M, D, R, S | ME+ SA | Md, Gr | Shrub | CH | NE |
| *Artemisia monosperma* Delile | Asteraceae | N, O, M, D, S | SA+ME | Md, Gr | Shrub | CH | NE |
| *Arthrocaulon macrostachyum* (Moric.) Piirainen & G.Kadereit | Amaranthaceae | N, O, M, De, R, S | ME+ SA | Gr, Md, Fu, Ot | Frut. | CH | NE |
| *Arundo donax* L. | Poaceae | N, O, M, D, S | ME+IT | Gr, Md, Fu | Per. | PH | LC |
| *Asparagus horridus* L. | Asparagaceae | O | ME+ SA | Md, Gr | Shrub | CH | NE |
| *Asphodelus aestivus* Brot. | Asphodelaceae | M, S | ME | Md, Hf, Ot | Per. | GH | LC |
| *Asphodelus tenuifolius* Cav. | Asphodelaceae | N, O, M, D, R, GE, S | ME+ SA+ SZ | Gr, Md, Fu, Orn, Ot | Ann. | TH | NE |
| *Astragalus boeticus* L. | Fabaceae | M, S | ME | Gr | Ann. | TH | NE |
| *Astragalus peregrinus* Vahl | Fabaceae | M, S | SA+ME | Gr, Me | Ann. | TH | NE |
| *Astragalus spinosus* (Forssk.) Muschl. | Fabaceae | M, D, S | SA+ IT+ME | Gr, Hf | Shrub | CH | NE |
| *Atractylis carduus* (Forssk.) C.Chr.var. *glabrescens* | Asteraceae | M, D, GE, S | SA+ME | Md | Per. | CH | NE |
| *Atractylis carduus* var. *marmarica* Täckh. & Boulos | Asteraceae | M, D, GE, S | Endemic | Md | Per. | CH | NE |
| *Atractylis prolifera*Boiss. | Asteraceae | M, S | SA+ME | Md | Ann. | TH | NE |
| *Atriplex halimus* L. | Amaranthaceae | M, D, S | SA+IT+ME | Gr, Md, Hf, Fu, Orn, Ot | Frut. | CH | LC |
| *Atriplex lindleyi* subsp. *inflata* (F.Muell.) Paul G.Wilson | Amaranthaceae | N, M, D | SA+ME | Gr, Md | Ann. | TH | NE |
| *Atriplex portulacoides* L. | Amaranthaceae | M, S | COSM | Gr | Per. | CH | NE |
| *Avena sterilis*L. | Poaceae | N, O, M, D, S | ME+ IT | Gr | Ann. | TH | LC |
| *Bassia muricata*(L.) Asch. | Amaranthaceae | O, M, D, S | SA+IT | Gr, Md, Fu | Ann. | TH | NE |
| *Bassia indica* (Wight) A.J.Scott | Amaranthaceae | N, O, M, D, S | SZ+ IT | Gr, Md, Hf, Fu | Ann. | TH | NE |
| *Bellevalia salaheidii* Täckh. & Boulos | Asparagaceae | S | Endemic | Md, Hf | Per. | GH | NE |
| *Bromus catharticus* Vahl | Poaceae | N, O, M, D | COSM. | Gr, Hf | Per. | HE | NE |
| *Bromus rubens* L. | Poaceae | M, O, D, S. | ME+IT+SA | Gr, Md | Ann. | TH | NE |
| *Bupleurum semicompositum* L. | Apiaceae | N, M, D, S | ME+ IT+ SA | Gr, Md, Ar, Fu, Hf, Ot | Ann. | TH | NE |
| **Cakile maritima* Scop. | Brassicaceae | N, M, D | ME+ES | Gr, Md, Ar, Ot | Ann. | TH | NE |
| **Calamagrostis arenaria* (L.) Roth | Poaceae | M, S | ME | Gr, Fu | Per. | GE | NE |
| *Calendula arvensis* L. | Asteraceae | N, O, M, D, S | ME+IT+SA | Gr, Md | Ann. | TH | NE |
| *Carduncellus eriocephalus* Boiss. var. *albiflora* | Asteraceae | M, D, S | SA+ME | Gr, Md | Per. | CH | NE |
| *Carduus getulus* Pomel | Asteraceae | N, M, D, M | SA+ME | Gr | Ann. | TH | NE |
| *Caroxylon tetragonum*(Delile) Moq. | Amaranthaceae | M, O, D | SA+ME | Md, Fu | Shrub | CH | NE |
| *Caroxylon tetrandrum*(Forssk.) Akhani & Roalson | Amaranthaceae | O, M, D, S | SA+ME | Md, Fu | Shrub | CH | NE |
| *Carrichtera annua*(L.) DC. | Asteraceae | N, M, D, S | SA+ME | Gr, Md, Orn | Ann. | TH | NE |
| *Cenchrus ciliaris* L. | Asteraceae | N, M, D, R, GE, S | PAL | Gr, Md | Per. | HE | LC |
| *Cenchrus echinatus*L. | Asteraceae | N, M | Neotropical | Gr | Ann. | TH | LC |
| *Centaurea alexandrina Delile* | Asteraceae | M | ME+SA | Gr, Md | Bien. | CH | NE |
| *Centaurea calcitrapa* L. | Asteraceae | N, O, M | ME+SA | Gr, Md, Hf | Ann.or short-lived Per. | TH | NE |
| *Centaurea dimorpha* Viv. | Asteraceae | M | ME+SA | Gr, Md | Per. | HE | NE |
| *Centaurea glomerata* Vahl. | Asteraceae | M, O, D | ME | Md, Gr | Ann. | TH | NE |
| *Chenopodium album* L. | Amaranthaceae | N, O, M, D, S | COSM | Md,Gr, Hf | Ann. | TH | NE |
| *Chenopodiastrum murale* (L.) S.Fuentes, Uotila & Borsch | Amaranthaceae | N, O, M, D, GE, S | COSM | Md,Gr, Hf | Ann. | TH | NE |
| *Chiliadenus candicans* (Delile) Brullo | Asteraceae | M, D | ME+SA | Md, Orn, Ot | Per. | CH | NE |
| *Coincya tournefortii* (Gouan) Alcaraz, T.E.Díaz, Rivas Mart. & Sánchez-Gómez | Brassicaceae | N., O., M., D., S. | ME+SA | Md, Gr | Ann. | TH | NE |
| *Convolvulus althaeoides* L. | Convolvulaceae | M, D, S | ME+ SA | Md, Gr | Per. | HE | NE |
| *Convolvulus arvensis* L. | Convolvulaceae | N, O, M, De, S | COSM | Md, Gr | Per. | HE | NE |
| **Crocodilium pumilio* (L.) N.Garcia & Susanna | Asteraceae | M | ME | Gr, Md | Ann. | TH | NE |
| **Crucianella maritima* L. | Rubiaceae | M, O, De, S | ME | Md | Per. | HE | NE |
| *Cutandia dichotoma*(Forssk.) Trab. | Poaceae | N, M, De, S | SA+ IT | GR | Ann. | TH | NE |
| *Cynanchum acutum* L. | Apocynaceae | N, O, M | ME + IT +ES | GR, Md, Fu, Ot | Per. | PH | LC |
| *Cynodon dactylon* (L.) Pers. | Poaceae | N, O, M, D, R, GE, S | TR | Md, Gr | Per. | GH | NE |
| *Dactyloctenium aegyptium* (L.) Willd.Poaceae | Poaceae | M, N, De, R, GE | PAL+ ME | Md, Hf, Ot | Per | TH | NE |
| **Daucus pumilus* (L.) Hoffmanns. & Link | Apiaceae | N, M, D | ME+SA | Ot | Ann. | TH | LC |
| **Daucus syrticus* Murb. | Apiaceae | M | ME | Md, Gr, Ar | Ann. | TH | NE |
| *Deverra tortuosa* (Desf.) DC. | Apiaceae | N, O, M, D, R, Si | SA+ME | Gr, Md, Hf, Fu, Ar, Ot | Per. | CH | NE |
| *Drimia maritima* (L.) Stearn | Asparagaceae | M, S | PAL | Md, Ot | Per. | GE | LC |
| *Echinops hussonii*Boiss. | Asteraceae | D, R, GE, M | SA+ME | Gr, Md, Hf | Per. | HE | NE |
| *Echinops spinosissimus* Turra | Asteraceae | N, M, D, R, GE, S | ME+SA+IT | Gr, Md, Fu, Hf | Per. | HE | NE |
| **Echinops taeckholmianus* Amin | Asteraceae | M | Endemic | Md, Gr | Per. | HE | EN |
| **Echiochilon fruticosum* Desf. | Boraginaceae | N, M, D, S | SA+ME | Gr, Ed, Fu | shrub | CH | NE |
| *Echium angustifolium* subsp*. sericeum (Vahl) Klotz* | Boraginaceae | M, D, S | ME | Md | Per. | CH | NE |
| *Enarthrocarpus lyratus* (Forssk.) DC. | Brassicaceae | N, O, M, D, S | ME | Gr, Md, Ar | Ann. | TH | NE |
| *Erodium crassifolium* L’ Hér. | Geraniaceae | M, D, S | SA +ME | Gr, Orn, Hf | Per. | CH | NE |
| *Erodium laciniatum* subsp. *laciniatum* | Geraniaceae | N, M, D, R, GE, S | SA+ ME | Gr, Md | Ann. | TH | NE |
| *Erodium laciniatum* subsp. *pulverulentum* | Geraniaceae | N, M, D, R, S | SA+ ME | Gr, Md | Ann. | TH | NE |
| *Eryngium campestre* L. | Apiaceae | M, S | ME+ IT | Md | Per. | HE | NE |
| *Eryngium creticum* Lam. | Apiaceae | M, S | ME+ IT | Md | Per. | HE | NE |
| *Euphorbia hirta* L. | Euphorbiaceae | N, M, S | SZ | Md, Ot | Ann. | TH | NE |
| **Euphorbia paralias* L. | Euphorbiaceae | M,S | ME+ ES | Md | Per. | HE | NE |
| *Euphorbia prostrata* Aiton | Euphorbiaceae | N, M, S | Neotropical | Md | Ann. | TH | CR |
| **Ficus palmata* Forssk. | Moraceae | De, GE, S | ME+ SA | Md, Hf | Tree | PH | LC |
| *Filago desertorum* Pomel | Asteraceae | M, D, R, S | SA+ IT | Gr | Ann. | TH | NE |
| *Frankenia hirsuta* L. | Frankeniaceae | O, M, D, S | ME+ IT+ ES | Md | Shrub | HE | NE |
| *Fumaria densiflora* DC. | Fumariaceae | N, O, M, D | ME+ES+IT | Gr | Ann. | TH | NE |
| *Fumaria parviflora* Lam. | Fumariaceae | N, M, D, S | ME+ES+IT | GR | Ann. | TH | NE |
| *Gastrocotyle hispida* (Forssk.) Bunge | Boraginaceae | N, M, D, S | IT+ SA | Md, Hf, Fu | Ann. | TH | NE |
| *Glebionis coronaria* (L.) Tzvelev | Asteraceae | N, M | SA+ME | Md, Hf, Orn | Ann. | TH | NE |
| *Globularia arabica* Jaub. & Spach | Plantaginaceae | M, D, S | SA+ME | Gr, Md, Fu | Shrub | CH | NE |
| *Gymnarrhena micrantha* Desf. | Asteraceae | M, D, S | IT+ SA | Gr | Ann. | TH | NE |
| *Gymnocarpos decander* Forssk. | Caryophyllaceae | M, D, S | SA+ME | Gr, Fu, Md | Per. | CH | NE |
| *Haloxylon salicornicum* (Moq.) Bunge ex Boiss. | Amaranthaceae | R, S, O, M, D | SZ+ME | GR, Md, OT | Shrub | CH | NE |
| *Helianthemum crassifolium* subsp. *sphaerocalyx*(Gauba & Janch.) Maire | Cistaceae | M | ME | Gr, Md | Frut. | CH | NE |
| *Helianthemum lippii (*L.) Dum.Cours. | Cistaceae | O, M, D, R, S | SA+ SZ | Gr, Md, Fu | Per. | CH | NE |
| *Helianthemum stipulatum* (Forssk.) C. Chr. | Cistaceae | M, De, S | ME+ SA | Gr, Md, Fu | Frut. | CH | NE |
| *Heliotropium digynum* (Forssk.) Asch. ex C.Chr. | Boraginaceae | N, M, D, R, S | SA | Md, Ar | Frut. | CH | NE |
| *Herniaria hemistemon* J. Gay | Caryophyllaceae | M, De, S | ME+SA | Gr, Md | Per. | CH | NE |
| *Herniaria hirsuta* L. | Caryophyllaceae | O, M, D, S | ES+ ME+ IT | Md | Ann. | TH | NE |
| *Hippocrepis areolata* Desv. | Fabaceae | M, De, S | ME+SA | Gr, Md | Ann. | TH | NE |
| *Hordeum marinum* subsp. *marinum* | Poaceae | N, O, M, S | ME+IT+ES | Md | Ann. | TH | NE |
| *Hordeum murinum* L. subsp*. galucum* (Steud.) Tzvelev | Poaceae | N, O, M, D, S | ME+IT | Gr, Md | Ann. | TH | LC |
| *Hordeum murinum* subsp*. leporinum* (Link) Arcang. | Poaceae | N, O, M, D, S | ME+IT | Gr, Md | Ann. | TH | LC |
| **Hyoseris radiata* subsp. *graeca* | Asteraceae | M | ME | Gr, Md | Per. | HE | NE |
| **Hyoseris scabra* L. | Asteraceae | M | ME | Gr, Md, Ot | Ann. | TH | NE |
| *Ifloga spicata* (Forssk.) Sch.Bip. | Asteraceae | N, M, D, R, GE, S | SA+ME | Gr | Ann. | TH | NE |
| *Imperata cylindrica* (L.) Raeusch. | Poaceae | N, O, M, D, R, S | PAL | Gr, Md, Ot | Per. | HE | NE |
| *Juncus hybridus* Brot. | Juncaceae | N, O, M, S | ME+ IT+ ES | Gr, Md | Ann. | TH | LC |
| *Juncus rigidus* Desf | Juncaceae | N, O, M, D, R, GE, S | ME+ IT+ ES | Gr, Md, Ot | Shrub | GH | LC |
| *Launaea capitata* (Spreng.) Dandy | Asteraceae | N, O, M, D, R, GE, S | SA+SZ+ME | Gr, Hf | Ann. or short-lived Per. | HE | NE |
| **Launaea fragilis* (Asso) Pau subsp. fragilis | Asteraceae | N, O, M, D, R, GE, S | SA+SZ+ME | Gr, Hf | Ann. | TH | NE |
| *Launaea mucronata* subsp*. cassiniana* (Jaub. & Spach) N.Kilian | Asteraceae | N, O, D, R, GE, S | ME + SA | Gr, Md | Ann. or short-lived Per. | TH | NE |
| *Launaea nudicaulis* (L.) Hook. F. | Asteraceae | N, O, M, D, R, GE, S | SA+ SZ+ IT | Gr, Md | Per. | HE | NE |
| *Lepidium coronopus* (L.) Al-Shehbaz | Brassicaceae | N, O, M | ME + IT+ ES | Ot | Ann. or Bien. | TH | LC |
| *Limbarda crithmoides*(L.) Dumort. | Asteraceae | N, O, M | ME+ ES+ SA | Md | Per. | CH | NE |
| *Limoniastrum monopetalum* (L.) Boiss*.* | Plumbaginaceae | M, S | ME | Gr, Fu | Shrub | CH | NE |
| *Limonium pruinosum* (L.) Chaz. | Plumbaginaceae | M, D, S | SA+ME | Gr, Md | Per. | HE | NE |
| **Lobularia libyca* (Viv.) Meisn. | Brassicaceae | M, D, S | SA | Gr, Md | Ann. | TH | NE |
| *Lobularia maritima* (L.) Desv. | Brassicaceae | N, M, S | ME | Gr, Md | Per. | CH | NE |
| *Lolium multiflorum* Lam. | Poaceae | N, O, M, D, R, S | ME+IT+ES | Gr, Md | Ann. or short-lived Per. | TH | LC |
| *Lolium perenne* L. | Poaceae | N, O, M, D, S | ES+ ME+ IT | Md, Hf | Per. | HE | LC |
| *Lotus arabicus* L. | Fabaceae | N, O, S | SZ+ SA | Md, Gr | Ann. | TH | LC |
| *Lotus creticus* L. | Fabaceae | M, D, S | ME | Gr, Md | Per. | CH | NE |
| **Lotus halophilus* Boiss. & Spruner | Fabaceae | N, O, M, D, S | ME+ SA | Gr, Md | Ann. | TH | NE |
| **Lotus polyphyllos* E.D.Clarke | Fabaceae | M | ME | Gr, Md | Per. | CH | NE |
| *Lotus tenuis* Waldst. & Kit. ex Willd. | Fabaceae | N, O, M | ES+ ME+ IT | Md, Gr | Per. | CH | NE |
| *Lycium europaeum* L. | Solanaceae | N, M, D | ME | Gr, Md, Fu, Hf, Ot | Shrub | PH | NE |
| *Lycium shawii* Roem. & Schult. | Solanaceae | N, M, D, R, GE, S | SA+ SZ | Hf, Ti, Gr, Md, Fu | Tree | PH | LC |
| *Lygeum spartum* Loefl. ex L. | Poaceae | M, S | SA+ ME | Ot (Manufacture) | Per. | GH | NE |
| *Malva ludwigii*(L.) Soldano, Banfi & Galasso | Malvaceae | N, O, M, De, S | SA+ME | Md | Ann. or Per. | CH or TH | NE |
| *Malva parviflora* L. | Malvaceae | N, O, M, D, R, S | ME+ IT | Gr , Md , Hf | Ann. | TH | NE |
| *Matricaria aurea* (Loefl.) Sch.Bip. | Asteraceae | N, M, D, S | ME+ IT | Md, Gr, Orn | Ann. | TH | NE |
| *Matthiola longipetala* subsp. *livida* (Delile) Maire | Brassicaceae | N, M, R, S | ME+ IT | Md | Ann. | TH | NE |
| *Medicago ciliaris*(L.) All. | Fabaceae | M | ME | Md, Gr, Ot | Ann. | TH | LC |
| **Medicago marina* L. | Fabaceae | M, S | ME | Ot | Per. | HE | LC |
| *Medicago polymorpha* L. | Fabaceae | N, M, O, D, S | ME+ IT+ ES | Md, Gr, Ot | Ann. | TH | LC |
| *Melilotus albus* Medik. | Fabaceae | N, M | ES+IT+ ME | Md, Gr | Ann. or Bien. | HE | LC |
| *Mesembryanthemum cryptanthum* Hook.f. | Aizoaceae | N, O, M, D | SZ+ME | Md, Hf | Ann. | TH | NE |
| *Mesembryanthemum crystallinum* L. | Aizoaceae | M, N, D | ME+ES | Md, Hf | Ann. | TH | NE |
| *Mesembryanthemum nodiflorum* L. | Aizoaceae | M, N, S | ME+ ES+ SA | Md, Hf | Ann. | TH |  |
| *Muscari albiflorum* (Täckh. & Boulos) Hosni | Asparagaceae | M | Endemic | Md, Hf | Per. | GE | NE |
| **Neurada procumbens* L. | Neuradaceae | O, M, D, R, GE, S | SA | Md | Ann. | TH | NE |
| *Nicotiana glauca* Graham. | Solanaceae | N, O, M, D, S | PAN | Md, Hf, Fu, Ot | Tree | PH | NE |
| *Nigella arvensis* L. | Ranunculaceae | M | ME+ IT+ ES | Md | Ann. | TH | NE |
| **Nitraria retusa* (Forssk.) Asch. | Nitrariaceae | N, O, M, D, R, GE, S | SA+ME | Md , Gr, Fu, Ot | Tree | PH | NE |
| *Noaea mucronata* (Forssk.) Asch. & Schweinf. | Amaranthaceae | M, D, S | ME+ IT | Md | Shrub | CH | NE |
| *Onobrychis crista-galli* (L.) Lam. | Fabaceae | M, D, S | IT+ SA+ME | Md, Gr | Ann. | TH | NE |
| *Ononis serrata* Forssk. | Fabaceae | M, D, S | ME+SA | Gr, Md | Ann. | TH | NE |
| **Ononis vaginalis* Vahl | Fabaceae | M | ME | Gr, Md, Fu | Shrub | CH | NE |
| *Onopordum alexandrinum* Boiss. | Asteraceae | M, S | ME | Md | Bien.or short-lived Per. | HE | NE |
| *Orobanche crenata* Forssk. | Orobanchaceae | N, M, De, S | ME+ IT | Ot | Ann. | TH | NE |
| **Pancratium arabicum* Sickenb. | Amarylidaceae | M,S | Endemic | Md | Per. | GH | EN |
| **Pancratium maritimum* L. | Amarylidaceae | M,S | ME | Md | Per. | GH | LC |
| *Panicum repens*  L. | Poaceae | N, O, M, D | PAL+ Neotropical + ME | Gr | Per. | CH | LC |
| *Parapholis marginata* Runemark | Poaceae | M, N, O, D, S | ME+IT+ES | Hf | Ann. | TH | NE |
| *Paronychia arabica* (L.) DC. | Caryophyllaceae | O, M | SA+ ME+SZ | Md, Hf | Ann. | TH | NE |
| *Paronychia argentea* Lam. | Caryophyllaceae | M, GE, S | ME | Md | Ann. or short-lived per. | HE | NE |
| *Paronychia capitata* subsp. *capitata* | Caryophyllaceae | M | ME | Md | Per. | CH | NE |
| *Phagnalon rupestre* (L.) DC. | Asteraceae | M, S | ME | Md | Frut. | CH | NE |
| *Phoenix dactylifera* L. | Arecaceae | N, O, M, D, R, GE, S | SA+SZ+ME | Md, Hf, Fu | Tree | PH | LC |
| *Phonus mareoticus* (Delile) G.López | Asteraceae | M, D | ME+IT | Gr, Md | Frut. | CH | NE |
| *Phragmites australis* (Cav.) Trin.ex Steud. | Poaceae | N, O, M, D, R, S | ME+ IT+ SA+ PAL+PAN | Gr , Md , Hf , Fu, Ot | Per. | GH | LC |
| **Picris asplenioides* L. | Asteraceae | N, M, D, S | ME+IT | Gr, Md | Ann. | TH | NE |
| *Pithecellobium dulce* (Roxb.) Benth | Fabaceae | N,M |  | Md, Fu | Tree or shrub | PH | LC |
| **Plantago afra* L. | Plantaginaceae | De, R, GE, S | ME+ IT | Md, Gr | Ann. | TH | LC |
| *Plantago albicans* L. | Plantaginaceae | M, S | ME+ SA | Md, Hf | Per. | HE | NE |
| *Plantago crypsoides* Boiss. | Plantaginaceae | M, D | ES+ME+IT | Md, Hf | Ann. | TH | NE |
| **Plantago indica* L. | Plantaginaceae | M, D, S, O, N | ME+SA | Md, Hf | Ann. | TH | NE |
| *Plantago notata* Lag. | Plantaginaceae | M, S | IT+SA | Md, Hf | Ann. | TH | NE |
| *Plantago ovata* Forssk. | Plantaginaceae | N, M, D, S | IT+SA | Gr, Md, Ar | Ann. | TH | LC |
| *Plantago phaeostoma*  Boiss. & Heldr. | Plantaginaceae | M, D, S | SA+ME | Md, Hf | Ann. | TH | NE |
| *Polygonum equisetiforme* Sm. | Polygonaceae | N, O, M, D, S | ME+IT | Md | Per. | HE | NE |
| *Pseudognaphalium luteoalbum* (L.) Hilliard & B. L. Burtt | Asteraceae | N, O, M, De, S | Cosm. | Md, Gr | Ann. | TH | LC |
| *Pseudopodospermum undulatum* (Vahl) Zaika, Sukhor. & N.Kilian | Asteraceae | N, D | ME+SA | Hf. Gr, Md | Per. | HE | NE |
| *Reaumuria hirtella* Jaub. & Spach | Tamaricaceae | M, D, S | SA+ IT | Md | Per. | CH | NE |
| *Reichardia tingitana* (L.) Roth | Asteraceae | N, M, D, R, GE, S | ME+IT | Md, Ot | Ann. | TH | NE |
| *Reseda alba* L. | Resedaceae | N, M, D, S | ME | Md | Ann. | TH | NE |
| *Reseda decursiva* Forssk. | Resedaceae | M, D, S | SA+ME | Gr, Md | Ann. | TH | NE |
| **Retama monosperma* (L.) Boiss. | Fabaceae | M | ME+ SA+ SZ | Gr, Fu, Md | Shrub | PH | NE |
| **Retama raetam* (Forssk.) Webb & Berthel. subsp. *raetam* | Fabaceae | M | SA+ IT | Gr, Fu, Md | Tree | PH | NE |
| **Rumex pictus* Forssk. | Polygonaceae | M, S | SA+ME | Md, Gr, Hf | Ann. | TH | NE |
| *Rumex spinosus* L. | Polygonaceae | N, O, M, D | SA +ME | Md, Gr, Hf, Fu | Ann. | TH | NE |
| **Saccharum spontaneum* L. | Poaceae | N,O, M, D, R, S | ME+ IT+ SA+ PAL | Md, Gr, Hf, Ot | Per. | GH | LC |
| *Salicornia fruticosa* (L.) L. | Amaranthaceae | N, O, M, D, S | ME+ES | Gr, Fu, Md | Frut. | CH | NE |
| *Salsola kali* L. | Amaranthaceae | N, M, S | COSM | Md , Hf , Ot | Ann. | TH | NE |
| *Salvia lanigera* Poir. | Lamiaceae | M, D, S | SA+ ME | Hf | Per. | CH | NE |
| *Salvia spinosa*subsp*. spinosa* | Lamiaceae | M, D, R, GE, S | SA+ SZ | Md, Hf, Orn | Frut. | TH | NE |
| *Salvia verbenaca* L. | Lamiaceae | M, D | SA+ ME | Md | Per. | HE | NE |
| *Schismus barbatus* (L.) Thell | Poaceae | N, O, M, D, R, S | ME + SA + IT | Hf | Ann. | TH | NE |
| *Senecio glaucus* subsp*. coronopifolius* (Maire) C. Alexander | Asteraceae | N, O, M, D, R, S | SA+ME | Hf, Md | Ann. | TH | NE |
| *Sesuvium portulacastrum* L. | Aizoaceae | M | SA+SZ+PAN | Eth | Per. | CH | LC |
| *Silene deversifolia* subsp*. bergiana* (Lindm.) L.Medina | Caryophyllaceae | N, M, S | ME | Ot | Ann. | TH | LC |
| **Silene succulenta* Forssk. | Caryophyllaceae | M | ME | Md, Ot | Per. | HE | NE |
| **Silene villosa* Forssk. | Caryophyllaceae | N, M, S | SA+IT+ME | Md | Ann. | TH | NE |
| *Sisymbrium irio* L. | Brassicaceae | N, M, D, GE, S | ME+ IT+ ES+ SA | Md, Gr, Hf | Ann. | TH | NE |
| *Soda longifolia*(Forssk.) Akhani | Amaranthaceae | M, D, S | SA+ ME | Md | Frut. | TH | NE |
| *Solanum elaeagnifolium* Cav. | Solanaceae | De, S | Tropical | Md, Ot | Per. | HE | NE |
| *Sonchus oleraceus* L. | Asteraceae | N, O, M, D, R, S | ME+ ES+ IT | Gr, Hf | Ann. | TH | NE |
| *Sonchus tenerrimus* L. | Asteraceae | M | ME+ IT+ ES+ SA | ME | Ann. | TH | NE |
| *Spergularia flaccida* (Madden) I.M.Turner | Caryophyllaceae | N, M, D, GE, S | SA+SZ+ME | Hf, Md | Ann. | TH | NE |
| *Spergularia marina* (L.) Besser | Caryophyllaceae | N, M | ES+ME+IT | Hf, Md | Ann. | TH | LC |
| *Spergularia media* (L.) C.Presl | Caryophyllaceae | M | ES+ME+IT | Hf, Md | Per. | CH | LC |
| *Sphenopus divaricatus* (Gouan) Rchb. | Poaceae | N, O, M, De, S | ME+ IT+ SA | Orn, Ot | Ann. | TH | LC |
| **Sporobolus pungens* (Schreb.) Kunth | Poaceae | M, S | ME | Gr, Ot | Per. | GE | NE |
| *Stellaria apetala* Ucria | Caryophyllaceae | N, O, M, S | SA+ ME | Md, Gr | Ann. | TH | NE |
| *Setaria pumila* (Poir.) Roem. & Schult. | Caryophyllaceae | N, O, GE, S | ME+ES | Gr | Ann. | TH | NE |
| *Stipellula capensis* (Thunb.) Röser & Hamasha | Poaceae | N, M, D, R, S | IT+ SA+ ME | Gr | Ann. | TH | NE |
| *Suaeda pruinosa Lange* | Amaranthaceae | M, S | SA+ SZ+ME | Gr, Fu | Shrub | CH | NE |
| *Symphyotrichum squamatum* (Spreng.) Nesom | Asteraceae | N, O, M, D, S | PAN | Ot | Ann. or Bien. | TH | NE |
| *Tamarix aphylla* (L.)H. Karst. | Tamaricaceae | N, O, M, D, R, GE, S | SA+ SZ+ME | Gr, Md, Fu, Ot | Tree | PH | LC |
| *Tamarix nilotica* (Ehrenb.) Bunge | Tamaricaceae | N, O, M, D, R, GE, S | SA+ IT | Gr, Fu, Ot | Tree | PH | LC |
| *Tamarix tetragyna* Ehrenb. | Tamaricaceae | N, O, M, D, R | ME+ SA | Gr, Fu, Ot | Tree | PH | LC |
| *Teucrium polium* L. | Lamiaceae | M, D, S | ME+ IT | Md | Per. | CH | NE |
| *Thesium humile* var. *maritima* Simps.(N.D. Simpson) Sa'ad | Santalaceae | M | Endemic | Md | Ann. | TH | NE |
| **Thinopyrum junceum*(L.) Á.Löve | Poaceae | N, M, S | ME | Gr | Per. | HE | LC |
| *Thymbra capitata* (L.) Cav. | Lamiaceae | M | ME+ SA | Md, Fu | Shrub | CH | LC |
| *Thymelaea hirsuta* (L.) Endl. | Thymelaeaceae | O, M, D, S | ME+ SA | Gr, Md, Fu, Ti, Ot | Shrub | PH | NE |
| *Traganum nudatum* Delile | Amaranthaceae | M, O, D, S | SA+ SZ+ME | Md, Ot, Fu | Shrub | CH | NE |
| *Trifolium tomentosum* L. | Fabaceae | M, D | ME+ IT+ ES | Md, Gr, Hf | Ann. | TH | NE |
| *Trigonella glabra* subsp. *glabra* | Fabaceae | N, O, M, S | SA+ SZ+ ME | Md | Ann. | TH | NE |
| *Urospermum picroides* (L.) F. W. Schmidt | Asteraceae | N, O, M, D, GE, S | ME+IT | Hf, Md | Ann. | TH | NE |
| *Vicia hirsuta* (L.) Gray | Fabaceae | D, M | PAL | Gr, Md | Ann. | TH | NE |
| *Vicia monantha* Retz. | Fabaceae | N, O, M, S | ME+ IT | Gr, Md | Ann. | TH | NE |
| *Volutaria lippii* (L.) Cass. ex Maire | Asteraceae | S, M, D | ME+ SA | Md | Ann. | TH | NE |
| *Zilla spinosa* (L.) Prantl | Brassicaceae | N, D, R, S | SA | Gr, Fu, Md | Shrub | PH | NE |
| *Zygophyllum aegyptium* Hosny | Zygophyllaceae | N, M, D, S | ME | Md | Frut. | CH | NE |
| *Zygophyllum album* L.f. | Zygophyllaceae | N, O, M, D, R, S | SA+ME | Gr, Md, Ot, Fu | Shrub | CH | NE |
| *Zygophyllum arabicum* (L.) Christenh. & Byng | Zygophyllaceae | O, M, D, S | ME+SA | Gr, Md, Fu | Frut. | CH | NE |
| *Zygophyllum creticum* (L.) Christenh. & Byng | Zygophyllaceae | M | SA | Gr, Md | Per. | CH | NE |
| *Zygophyllum indicum* (Burm.f.) Christenh. & Byng | Zygophyllaceae | O, D, R, GE, S, M | SA | Gr, Md | Per. | CH | NE |
| *Zygophyllum simplex* L. | Zygophyllaceae | D, R, GE, S, M | SA | Md, Ot | Ann.or short-lived Per. | TH | NE |

**Appendix 3** Land cover change from 1988 to 2070 in the Mediterranean coastal strip region in Egypt.

| **Class name** | **1988** | | **2024** | | **2050** | | **2070** | |
| --- | --- | --- | --- | --- | --- | --- | --- | --- |
|  | **Area (km^2^)** | **%** | **Area (km^2^)** | **%** | **Area (km^2^)** | **%** | **Area (km^2^)** | **%** |
| **Agriculture** | 1195.6 | 11.6 | 1240.4 | 12.1 | 1565.1 | 15.2 | 1678.6 | 16.3 |
| **Bare Land** | 7785.3 | 75.7 | 6763.5 | 65.8 | 5612.7 | 54.5 | 4413.5 | 42.9 |
| **Fish farm** | 61.2 | 0.59 | 565.0 | 5.5 | 521.1 | 5.1 | 560.3 | 5.5 |
| **Urban** | 203.5 | 1.98 | 712.4 | 6.9 | 1573.9 | 15.3 | 2603.6 | 25.3 |
| **Vegetation** | 373.8 | 3.64 | 257.9 | 2.5 | 100.1 | 0.97 | 90.0 | 0.88 |
| **Water** | 652.8 | 6.35 | 740.7 | 7.2 | 907.0 | 8.8 | 933.8 | 9.1 |
